# The Dpp/TGFβ-Dependent Corepressor Schnurri Protects Epithelial Cells from JNK-Induced Apoptosis in *Drosophila* Embryos

**DOI:** 10.1016/j.devcel.2014.08.015

**Published:** 2014-10-27

**Authors:** Jorge V. Beira, Alexander Springhorn, Stefan Gunther, Lars Hufnagel, Giorgos Pyrowolakis, Jean-Paul Vincent

**Affiliations:** 1 Medical Research Council National Institute for Medical Research, The Ridgeway, Mill Hill, London NW7 1AA, UK; 2 Department of Cell and Developmental Biology, University College London, Gower Street, London WC1E 6BT, UK; 3 BIOSS Centre for Biological Signalling Studies and Institute for Biology I, University of Freiburg, Schänzlestrasse 18, 79104 Freiburg, Germany; 4 Spemann Graduate School of Biology and Medicine, Research Training Program GRK 1104, Albert Ludwigs University, 79104 Freiburg, Germany; 5 European Molecular Biology Laboratory, Meyerhofstrasse 1, 69117 Heidelberg, Germany

## Abstract

Jun N-terminal kinase (JNK) often mediates apoptosis in response to cellular stress. However, during normal development, JNK signaling controls a variety of live cell behaviors, such as during dorsal closure in *Drosophila* embryos. During this process, the latent proapoptotic activity of JNK becomes apparent following Dpp signaling suppression, which leads to JNK-dependent transcriptional activation of the proapoptotic gene *reaper.* Dpp signaling also protects cells from JNK-dependent apoptosis caused by epithelial disruption. We find that repression of *reaper* transcription by Dpp is mediated by Schnurri. Moreover, reporter gene analysis shows that a transcriptional regulatory module comprising AP-1 and Schnurri binding sites located upstream of *reaper* integrate the activities of JNK and Dpp. This arrangement allows JNK to control a migratory behavior without triggering apoptosis. Dpp plays a dual role during dorsal closure. It cooperates with JNK in stimulating cell migration and also prevents JNK from inducing apoptosis.

## Highlights

• Dpp/BMP signaling prevents JNK from activating expression of proapoptotic *reaper*
• The transcriptional repressor Schnurri mediates the antiapoptotic effect of Dpp
• AP-1 and Schnurri binding sites integrate the inputs of JNK and Dpp on *reaper*
• Dpp signaling ensures the survival of dorsal edge cells during dorsal closure

## Graphical Abstract

**Figure:**
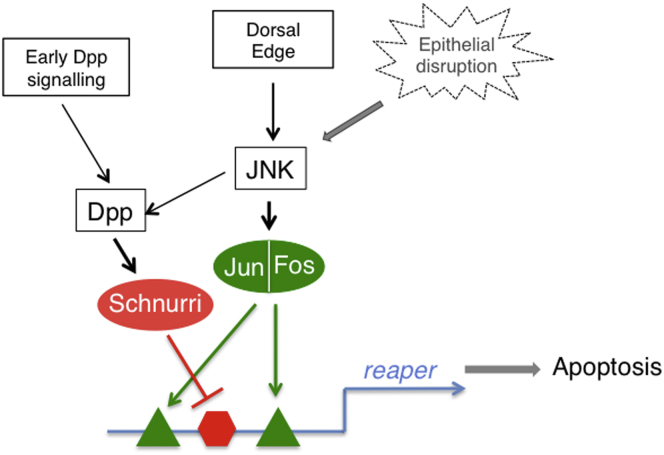

## Teaser

JNK often mediates apoptosis in response to cellular stress, yet JNK signaling controls a variety of live cell behaviors during normal development. In *Drosophila* embryos, JNK can direct dorsal closure because concurrent Dpp signaling, mediated by the repressor Schnurri, prevents activation of the proapoptotic gene *reaper* by JNK.

## Introduction

Signaling by c-Jun N-terminal kinase (JNK) mediates one of the major stress response pathways (Chen, 2012, Stronach and Perrimon, 1999). Indeed, activation of JNK signaling often boosts or triggers apoptosis (Dhanasekaran and Reddy, 2008, Igaki, 2009, Leppä and Bohmann, 1999). JNK can exert its proapoptotic effect through phosphorylation of Jun, a component of the AP-1 transcriptional activator, or of other cellular proteins (Bogoyevitch and Kobe, 2006). It is important to note, however, that JNK signaling does not always trigger apoptosis (Weston and Davis, 2007) and has been shown to control nonapoptotic processes such as cytoskeletal rearrangements (Homsy et al., 2006), cell migration (Ríos-Barrera and Riesgo-Escovar, 2013), and cell proliferation (Shaulian and Karin, 2002) during development and regeneration. It is generally thought that the cellular context or the activity of other signaling pathways determines whether JNK signaling leads to apoptosis or not. In well-documented instances, this involves downregulation or blunting of JNK signaling itself, e.g., through the activity of Gadd45β, an NF-κB-induced factor (De Smaele et al., 2001, Papa et al., 2004), or by Puckered, a feedback inhibitor of JNK signaling (McEwen and Peifer, 2005). Mechanisms that dampen JNK’s proapoptotic influence without affecting core pathway activity have also been documented. For example, in the developing *Drosophila* eye, mitogen-activated protein kinase phosphorylates and destabilizes Hid, a proapoptotic protein transcriptionally activated by JNK signaling in this tissue (Bergmann et al., 1998). Another documented process involves transcriptional repression of *hid*, which would otherwise be overactivated by JNK in response to irradiation damage (Luo et al., 2007). Non-cell-autonomous protective mechanisms could also be at work. For example, the transcriptional modulator Schnurri limits radiation-induced tissue damage by recruiting macrophages through activation of the PDGF-related growth factor Pvf1 (Kelsey et al., 2012). All the aforementioned mechanisms have been shown to operate in response to cellular stress. However, so far, little is known about the regulatory processes that prevent JNK from causing apoptosis during normal development. One well-characterized nonapoptotic JNK-dependent developmental process is the morphogenetic movement of dorsal closure (Glise and Noselli, 1997, Hou et al., 1997, Riesgo-Escovar and Hafen, 1997). Here, we set out to investigate the molecular mechanisms that prevent JNK from activating apoptosis during this process.

**Figure 1:**
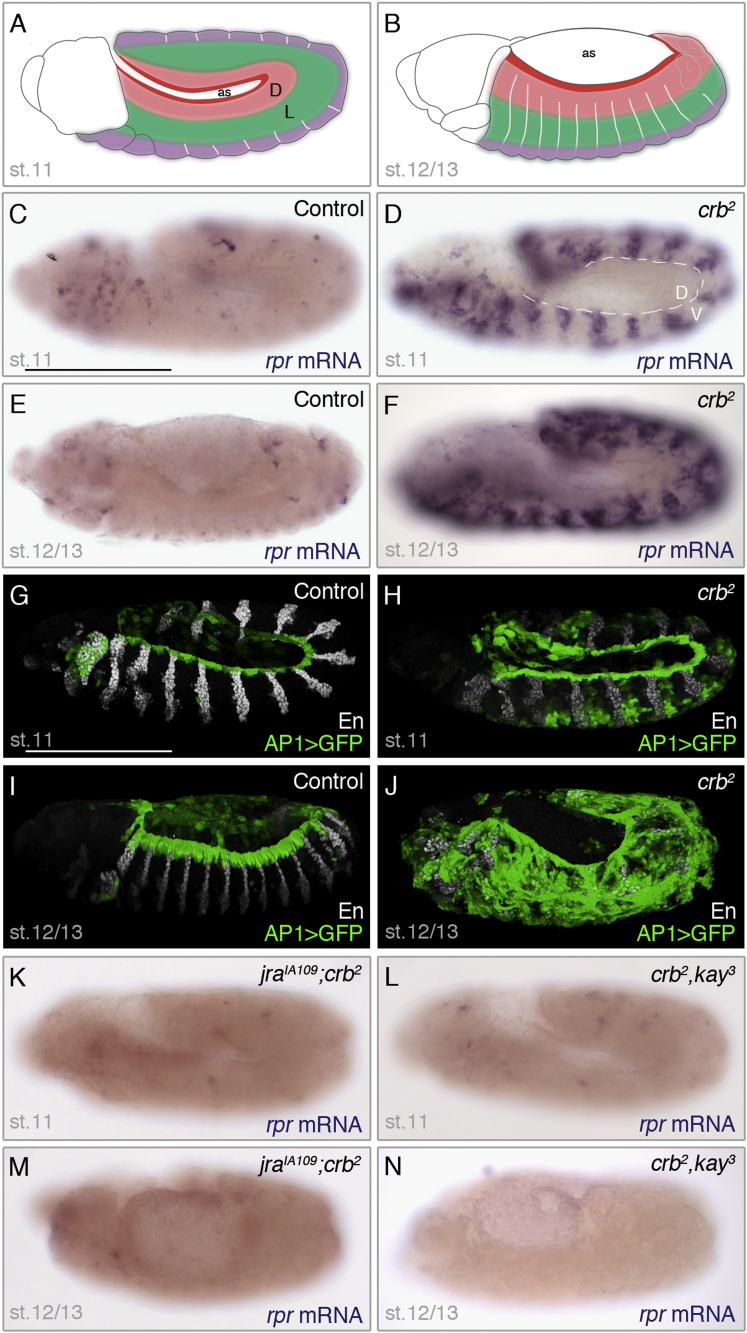
Epithelial Disruption Triggers Canonical JNK Signaling, which, in Turn, Activates *reaper* Transcription in the Ventral and Lateral Epidermis of *Drosophila* Embryos (A and B) Diagram of *Drosophila* embryos at stage (st.) 11 and stage 12/13, indicating the key epidermal domains: the dorsal (D) edge (deep red), the dorsal epidermis (faded red), the lateral (L) epidermis (green), and the ventral epidermis (purple). as, amnioserosa. (C–F) Expression of *reaper* in control (*crb*^*2*^*/+*) and *crumbs* mutant (*crb*^*2*^*/ crb*^*2*^) embryos at stages 11 (C and D) and 12/13 (E and F), as indicated. In homozygous *crumbs* mutants, segmental upregulation is seen in the ventrolateral (V) epidermis, while in the dorsal (D) epidermis (inside white dotted lines), it remains silent. mRNA, messenger RNA. (G–J) A fluorescent reporter of JNK activity (Chatterjee and Bohmann, 2012) is active at the edge of the dorsal epidermis of control embryos (*crb*^*2*^*/+*) and throughout the epidermis of homozygous *crumbs* mutant embryos (*crb*^*2*^*/ crb*^*2*^). Engrailed (En) immunoreactivity provides positional landmarks along the anterior-posterior axis. Embryonic stages are indicated. (K–N) Expression of *reaper* is not activated in *crumbs* mutant embryos that also lack *jra* (jun) or *kayak* (fos). As positive controls, *crumbs* mutant embryos were stained in parallel (data not shown). Genotypes and stages are indicated. Scale bar, 200 μm.

Dorsal closure is a morphogenetic movement that closes a large gap left on the embryo’s dorsal side after germ band retraction. It involves the concerted movement of the dorsal epidermis toward the midline and requires both JNK and Dpp signaling. One current view is that JNK signaling at the leading edge promotes expression of Decapentaplegic (Dpp, a transforming growth factor β [TGF-β] homolog), which, in turn, orchestrates the cell shape changes required for dorsal closure (Fernández et al., 2007, Ríos-Barrera and Riesgo-Escovar, 2013). It is likely that a protective mechanism is at work at the leading edge since JNK does not trigger apoptosis there. Additional evidence that the dorsal epidermis is protected from apoptosis came from the analysis of *crumbs* (*crb*) (abbreviated as *crb* in genotypic descriptions) mutants, where JNK target genes are upregulated in response to loss of apicobasal polarity (Kolahgar et al., 2011). In such embryos, most epidermal cells undergo apoptosis, except in an approximately ten-cell-wide band of dorsal cells, despite strong activation of JNK signaling there. It appears, therefore, that the protective mechanism acts over a broader domain than just within the leading edge (see Figures 1A and 1B for a diagram of the relevant region of the embryonic epidermis).

Most apoptosis in *Drosophila* requires the *H99* locus (White et al., 1994), which comprises the three main proapoptotic genes: *reaper*, *hid*, and *grim*. Among these, *reaper* is the most likely mediator of the response to epithelial disruption since it is upregulated in *crb* mutant embryos in a pattern prefiguring that of caspase immunoreactivity (Kolahgar et al., 2011). Moreover, overexpression of Puckered, a phosphatase that inhibits JNK signaling prevents *reaper* upregulation, as well as apoptosis, in *crb* mutants (Kolahgar et al., 2011). These observations suggested that loss of apicobasal polarity triggers JNK signaling (through an unknown mechanism), which, in turn, causes *reaper* expression and, hence, apoptosis. However, in the dorsal epidermis, JNK signaling does not activate *reaper* expression. Here, we show the molecular mechanism underpinning such protection and thus explain how JNK can control cell migration without triggering apoptosis.

## Results and Discussion

### Activation of *reaper* by Canonical JNK Signaling Mediates Apoptosis in Response to Epithelial Disruption

In *crb* mutant embryos, *reaper* is strongly upregulated (Figures 1C–1F) in a pattern similar to that of apoptosis (Figures S1A and S1B available online), while the other two main proapoptotic genes, *hid* and *grim*, remain largely silent (Kolahgar et al., 2011)). No activated caspase immunoreactivity was detectable in *rpr*^*87*^ *crb* double mutant embryos (Figure S1C), confirming the essential role of *reaper* and highlighting the need to uncover the mechanisms that activate *reaper* expression following loss of apicobasal polarity. As suggested previously (Kolahgar et al., 2011), JNK signaling is likely involved. Indeed, JNK signaling, as measured with a transcriptional reporter (AP-1 > GFP) (Chatterjee and Bohmann, 2012) was strongly activated in *crumbs* mutant embryos (Figures 1G–1J; Movie S1). Moreover, little *reaper* transcription was detectable in *crumbs* embryos that also lack *jra* or *kayak*, which encode the two components of AP-1, Jun, and Fos (Figures 1K–1N; see Figures S1F–S1I for *reaper* expression in the single mutants), and this was associated with a near-absence of apoptosis, as reported by activated caspase-3 immunoreactivity (Figures S1D and S1E). Notably, JNK signaling does not seem to necessarily cause apoptosis. In the ventral epidermis, the patterns of *reaper* expression and apoptosis (highlighted with anti-activated caspase) appeared to mirror the early segmental activation of JNK signaling (compare Figure 1D with Figure S1B), suggesting a relatively straightforward, likely causal, relationship there. However, neither *reaper* expression nor apoptosis were significantly activated in the dorsal epidermis, even at the dorsal edge, where JNK signaling is particularly active both in wild-type and *crumbs* mutants. What is the mechanism that protects the dorsal epidermis from the proapoptotic effect of JNK?

### Dpp Signaling Prevents JNK from Activating *reaper* Expression in the Dorsal Epidermis

**Figure 2:**
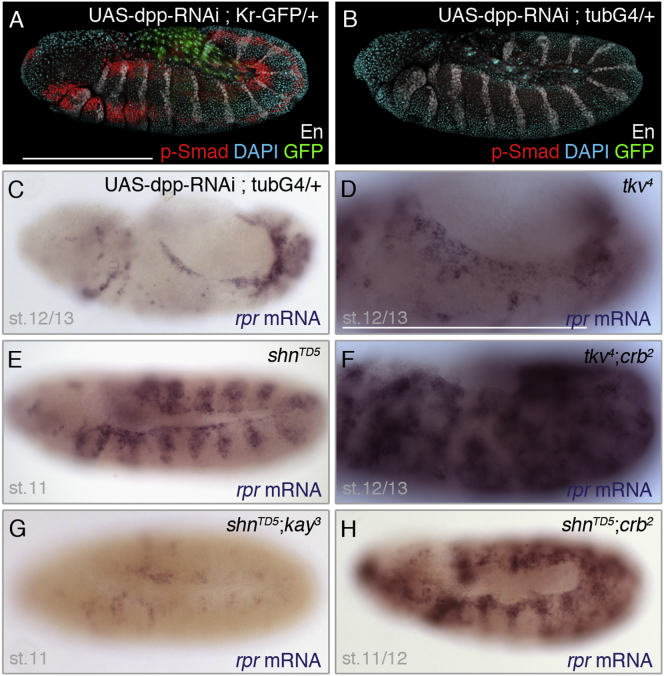
Dpp Signaling Counteracts JNK-Dependent Activation of *reaper* via the Transcriptional Repressor Schnurri (A and B) p-Smad immunoreactivity, seen in control embryos (A), is not detectable in embryos carrying both UAS-dpp[RNAi] and *tubulin-Gal4* (B), confirming the effectiveness of this RNAi transgene. Anti-Engrailed and DAPI were used as morphological landmarks. En, Engrailed; st., stage. (C) In embryos expressing UAS-dpp[RNAi] under the control of *tubulin-Gal4*, *reaper* expression becomes upregulated at the dorsal edge of the epidermis. Such embryos are often misshapen probably because of interference with the patterning activity of Dpp. (D) Expression of *reaper* is also upregulated at the dorsal edge of homozygous *thickveins (tkv)* mutants. (E) Upregulation of *reaper* in the dorsal and lateral epidermis of *schnurri* mutants. Expression can be seen along the whole dorsal edge but is segmental elsewhere. The basis of the segmental pattern is unknown. (F) Upregulation of *reaper* throughout most of the epidermis of *thickveins crumbs* double mutants. (G) No *reaper* upregulation is detected in *schnurri kayak (fos)* double mutants, demonstrating the requirement for canonical JNK signaling in *reaper* activation. (H) Expression of *reaper* is upregulated throughout the epidermis of *schnurri crumbs* double mutants in a pattern that is roughly the sum of those seen in the single mutants. Scale bar, 200 μm.

One feature of the dorsal epidermis is that it is under the influence of Dpp, a member of the BMP family of secreted growth factors (Hamaratoglu et al., 2014). Indeed, phospho-Smad (p-Smad) immunoreactivity (a mark of Dpp signaling) (Tanimoto et al., 2000) was detectable in this region before and during the time when JNK is active (Figure 2A). Therefore, Dpp signaling could prevent JNK signaling from activating *reaper* expression both in the dorsal epidermis of *crumbs* mutant embryos and, physiologically, at the dorsal edge of wild-type embryos. This was tested in embryos expressing a Dpp RNA interference (RNAi)-encoding transgene (Supplemental Information) under the control of the ubiquitous *tubulin-gal4* driver. This led to reduced signaling as indicated by the loss of p-Smad immunoreactivity from stage 11 onward (Figure 2B). In these embryos, a band of *reaper* transcription was observed at the dorsal edge (Figure 2C), where JNK is known to be activated in the wild-type (see Figure 1I). A similar result was seen in embryos lacking zygotic (but not maternal) activity of *thickveins* (*tkv*), which encodes an essential Dpp receptor (Nellen et al., 1994) (Figure 2D). Expression of *reaper* was also seen in the approximately ten-cell-wide dorsal region in *tkv crumbs* double mutants (as well as in the rest of the epidermis; Figure 2F), suggesting that Dpp signaling prevents *reaper* expression throughout the dorsal epidermis. Caspase immunoreactivity became detectable throughout the epidermis of *tkv crumbs* mutants (Figure S1J), consistent with the notion that repression of *reaper* expression by Dpp signaling translates into anti-apoptotic activity.

### Schnurri Prevents *reaper* Transcription and Apoptosis

Dpp signaling is mediated by the Mad complex, which can activate or repress target genes depending on the sequence context and other cofactors (Affolter and Basler, 2007). The best characterized instance of repression by Dpp signaling is that of the *brinker* gene, which occurs via silencer elements where the Mad complex recruits the corepressor Schnurri (Affolter and Basler, 2007, Pyrowolakis et al., 2004). We therefore asked if Schnurri could mediate the repression of *reaper* by Dpp. Indeed, *reaper* transcription was upregulated at the dorsal edge of *schnurri* mutant embryos (Figure 2E; Figures S1O–S1R). In these embryos, *reaper* expression was also seen to extend segmentally in the lateral region, a feature not seen in Dpp-RNAi-expressing embryos (compare Figure 2E with Figure 2C), perhaps because Schnurri also has Dpp-independent activity, as suggested by (Kelsey et al., 2012). In any case, the *reaper*-repressive role of Schnurri in the dorsal epidermis was confirmed in *schnurri crumbs* double mutants, where *reaper* transcription was strongly upregulated in both the dorsal and ventral regions (Figure 2H). No *reaper* upregulation was seen in *schnurri kayak (fos)* double mutants (Figure 2G), showing that JNK signaling is an essential positive input to *reaper* transcription at the dorsal edge, as well as in segmentally repeated lateral domains, where the JNK sensor appears not to be sufficiently sensitive to detect activity. It is worth noting that staining of *schnurri* and *schnurri crumbs* embryos with anti-activated caspase confirmed that the repression of *reaper* transcription by Schnurri is needed to suppress apoptosis (Figures S1K and S1L). As AP-1 and Schnurri are both transcriptional regulators, we next sought to address whether the opposing influences of Schnurri and JNK signaling converge directly on the *reaper* promoter.

### Binding Sites Upstream of the *reaper* Promoter Integrate the Effects of Dpp and JNK

**Figure 3:**
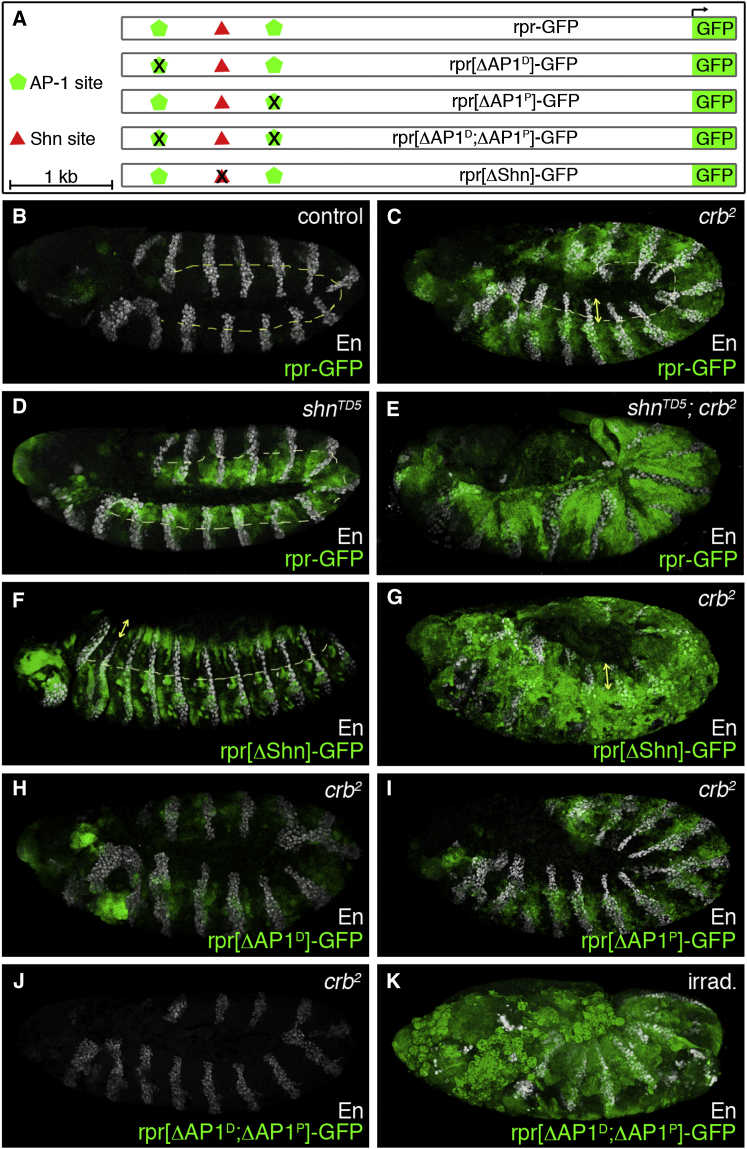
The *reaper* Promoter Integrates Inputs from JNK and Dpp Signaling (A) Diagram of the reporter constructs that were tested in transgenic embryos; 5.5 kb of the *reaper* promoter were used to create *rpr-GFP*. Predicted binding sites for Schnurri or AP-1 were mutated to generate the variants listed. Details of the mutations are indicated in the text. (B) The unmutated reporter (*rpr-GFP*) is almost silent in wild-type embryos. En, Engrailed. (C) In *crumbs* mutant embryos, the same reporter is active in the ventrolateral, but not the dorsal (double-headed arrow), epidermis. (D) By contrast, in *schnurri* mutants, *rpr-GFP* becomes active in the dorsal epidermis, suggesting that Schnurri is an essential repressive factor. (E) Embryos lacking both *crumbs* and *schnurri* upregulate *rpr-GFP* throughout much of the epidermis. (F) Mutation of the predicted Schnurri binding site leads to upregulation of the reporter in the dorsal epidermis wild-type embryos at stage 13. (G) In *crumbs* mutant embryos, *rpr[ΔShn]-GFP* upregulation is seen in the dorsal epidermis (double-headed arrow) as well as in the ventrolateral epidermis, consistent with the notion that Schnurri contributes to preventing *reaper* expression in the dorsal epidermis of *crumbs* embryos. (H–J) Upregulation of the *reaper* reporter in *crumbs* mutants requires the two predicted AP-1 binding sites. Deletion of either site leads to reduced expression while the double mutant reporter (*rpr[ΔAP1*^*D*^*; ΔAP1*^*P*^*]-GFP*) is silent. (K) Gamma-irradiated embryos carrying the *rpr[ΔAP1*^*D*^*; ΔAP1*^*P*^*]-GFP* transgene express GFP throughout, showing that this reporter is functional. All embryos are shown at stage 11 except for (F), which shows a stage 12–13 embryo. Engrailed (En), shown in white, provides an indication of the embryos’ overall morphology.

The promoter region of *brinker* that mediates Schnurri-dependent repression has been extensively characterized, and mutation analysis identified an essential 16-base-pair (bp) repressor sequence (Pyrowolakis et al., 2004). Similar elements are found at ∼350 positions in the *Drosophila* genome, defining a consensus sequence: GRCGNCNNNNNGTCTG (Pyrowolakis et al., 2004). Two related sites were identified upstream of *reaper*, in a region that is conserved in the 12 sequenced *Drosophila* species. In all these species, the proximal site (SE_p_) is flanked on either side by a predicted AP-1-binding site, making it a potential regulatory element. Because SE_p_ is not an exact match to the consensus Schnurri binding site, we used an electrophoretic mobility shift assay (EMSA) to test whether it is recognized by Schnurri, using the previously characterized site from the *brinker* gene (cSE) (Pyrowolakis et al., 2004) as a positive control. Recombinant Schnurri protein induced a supershift of the Mad-Medea-DNA complex in both cases, although to a lesser extent with SE_p_ than with cSE (Figure S2A). Thus, we conclude that SE_p_ is recognized by Schnurri/Mad/Medea and could therefore mediate the repressive influence of Dpp on *reaper* expression. By extension, the module comprising SE_p_ and the two putative AP-1 sites could integrate the influence of Dpp and JNK signaling on *reaper* expression. To test this hypothesis in vivo, we made a reporter construct comprising 5.5 kb of sequence including this module and the basal *reaper* promoter, upstream of a GFP complementary DNA (Figure 3A). This reporter (*rpr-GFP*) and the variants described later were introduced by PhiC31-mediated integration at the same genomic location to allow comparison without confounding influence from position effects. The wild-type reporter was essentially silent in wild-type embryos (Figure 3B), as expected, since *reaper* expression is barely detectable during normal embryogenesis. By contrast, in *crumbs* mutants, *rpr-GFP* became segmentally upregulated in the ventrolateral—but not dorsal—epidermis (double-headed arrow in Figure 3C), thus mirroring the activity of the endogenous *reaper* gene in this background (compare to Figure 1F). Critically, like the endogenous *reaper* gene, this reporter became active in the dorsal epidermis of *schnurri* mutants (Figure 3D). Such dorsal expression was segmentally modulated (see Figure S2B for GFP staining alone), resembling the pattern of endogenous *reaper* transcription in *schnurri* mutants (Figure 2E). Also, like endogenous *reaper*, the reporter was widely and strongly activated in the epidermis of *schnurri crumbs* double mutants (Figure 3E). These observations suggest that Schnurri mediates repression of the reporter. To test the contribution of the predicted Schnurri binding site, a mutation was introduced in the reporter (ATCGTCTCGCCGTCTG → ATCGTCTCGCTTTCTG), thus creating *rpr[ΔShn]-GFP* (Figure 3A). This mutation was found to abrogate formation of the Mad-Medea-DNA complex in vitro (see SEm in Figure S2A), suggesting that *rpr[ΔShn]-GFP* would no longer be subject to repression by Schnurri. Indeed, this transgene became activated in wild-type embryos in the dorsal epidermis (Figure 3F). One must point out that this dorsal activity of *rpr[ΔShn]-GFP* did not appear before stage 13, 1–2 hr later than the appearance of *rpr-GFP* in *schnurri* mutants (compare Figure 3F to Figure 3D). Also, unlike *rpr-GFP* in *schnurri* mutants, *rpr[ΔShn]-GFP* was active in segmentally repeated ventral domains at stage 13. Despite these differences (see further discussion in the legend of Figure S2), the upregulation of *rpr-GFP* in *schnurri* mutants and the expression of *rpr[ΔShn]-GFP* in the dorsal epidermis of otherwise wild-type embryos (where *rpr-GFP* is silent) are consistent with the notion that Schnurri represses *reaper* expression in the dorsal epidermis, thus allowing JNK signaling to control epithelial migration without triggering apoptosis.

We next assessed the function of the two predicted AP-1 binding sites by mutating them (TGACTCATA → TGACATTTA) (Chatterjee and Bohmann, 2012) individually or as a pair in the GFP reporter (Figure 3A) and assessing their activity in the ventral epidermis of *crumbs* embryos, where the wild-type reporter is strongly activated. Mutating one or the other site reduced activation, while the double mutant reporter (*rpr[ΔAP1*^*P*^*;ΔAP1*^*D*^*]-GFP*) had no detectable activity in *crumbs* mutants (Figures 3H–3J; wild-type background in Figure S2E). This reporter is nevertheless functional. It was activated by irradiation (Figure 3K), consistent with the presence of a p53-response element previously shown to mediate the response to irradiation (Brodsky et al., 2000). It is worth pointing out here that *reaper* expression was still activated in *crb p53*^*5A-1-4*^ double mutants (P.F. Langton and J.-P.V., unpublished data), indicating that the response to loss of epithelial integrity does not require p53. Overall, our results show that the two predicted AP-1 binding sites contribute redundantly to *reaper* upregulation in *crumbs* mutants. They also show that JNK signaling acts directly on the *reaper* promoter and not via a relay mechanism. We conclude that a small regulatory module allows JNK to trigger apoptosis, except in dorsal cells that are protected by Dpp signaling.

### Schnurri Ensures the Survival of Dorsal Edge Cells during Dorsal Closure

**Figure 4:**
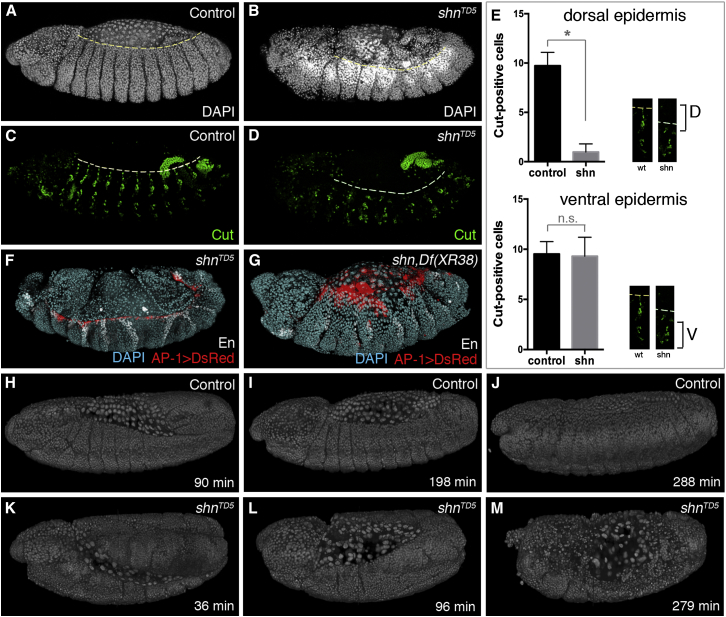
Dorsal Closure and Epithelial Loss in Wild-Type and *schnurri* Mutant Embryos (A and B) Homozygous *schnurri* mutant embryos fail to achieve dorsal closure. A heterozygous embryo of similar stage is shown as control. (C and D) The reduced number of Cut-positive cells in the dorsal cluster of *schnurri* mutant embryos suggests that the dorsal epidermis shrinks during the time when dorsal closure takes place in control embryos. (E) Number of Cut-positive cells in the dorsal (D) and ventral (V) clusters of *schnurri* homozygotes and control heterozygotes at stage ∼13. A significant loss of cells is seen in the dorsal cluster (Student’s t test, ^∗^p < 0.0001). WT, wild-type; n.s., not significant. Error bars indicate SEM. (F and G) *schnurri* and *schnurri reaper* mutant embryos carrying the JNK reporter. Gaping of dorsal hole is more pronounced in *schnurri* mutant (F) than in *schnurri reaper* mutant (G). En, Engrailed. (H–M) Still images from MuVi-SPIM recordings (before, during, and after closure). Approximate times from the beginning of germband retraction are shown to allow comparison between the mutant and control samples. Control (H–J) and *schnurri* mutant (K–M) embryos carrying histone-RFP as a nuclear marker are shown. While dorsal cells migrate over the amnioserosa in the wild-type, these cells are progressively lost in *schnurri* mutants. See also Movies S3 and S4.

JNK and Dpp signaling have extensively been shown to orchestrate dorsal closure. So far, attention has been focused on the role of these pathways in triggering the cell shape changes required for the dorsal epidermis to spread over the amnioserosa and meet at the dorsal midline (Fernández et al., 2007, Homsy et al., 2006, Riesgo-Escovar et al., 1996). Our results suggest an additional role for Dpp signaling during dorsal closure, namely, to ensure the survival of leading edge cells. We propose that such a protective mechanism is needed because of the proapoptotic influence of JNK signaling. Accordingly, the “dorsal open phenotype” of *schnurri* mutants (and possibly other Dpp pathway mutants) would not only be caused by the failure of dorsal edge cells to migrate but also by their reduced survival. To evaluate the contribution of the latter, we assessed the extent of tissue loss in fixed and live *schnurri* mutant embryos, which, as previously reported (Arora et al., 1995, Grieder et al., 1995, Staehling-Hampton et al., 1995), do not undergo dorsal closure. Cursory observation suggests that, in *schnurri* mutant embryos, the dorsal hole that appears at the end of germband retraction gapes open over time (Figures 4A and 4B; Figures S1Q and S1R). This was confirmed by confocal live imaging of *schnurri* mutants carrying *spider-GFP* as a marker of cell outlines (Figure S3D; Movie S2). To test if gaping of the dorsal hole could be due to tissue shrinkage, stage ∼13 *schnurri* mutant embryos were stained with anti-Cut, which marks dorsal and ventral clusters of peripheral neurons along the D-V axis (Figures 4A–4D; Figures S3A–S3C), and the number of Cut-positive cells in each cluster was counted. While cell number in the dorsal cluster remained constant in wild-type embryos, it decreased significantly in *schnurri* mutants (Figure 4E). The number of ventral cells was relatively unaffected in both genotypes. It appears, therefore, that the dorsal epidermis is preferentially eliminated prior to this stage. Next, we used multiview light-sheet microscopy (MuVi-SPIM; Krzic et al., 2012) to achieve in toto imaging of *schnurri* mutants and control embryos carrying histone-red fluorescent protein (histone-RFP) (Figures 4H–4M). In *schnurri* mutants, cell debris could be seen around the dorsal edge, and many macrophages scurried around (Movies S3 and S4). Moreover, the number of epidermal nuclei became reduced, compared to that in control embryos (Figure S4). These observations confirm that, during stages 12–14, the dorsal epidermis of *schnurri* mutants progressively shrinks (Figures S4A–S4I), likely by apoptosis (see also Figures S1K, S1Q, and S1R). Consistent with the involvement of apoptosis, the dorsal hole did not appear to gape open in *schnurri* mutants that also lacked *reaper* (Figure 4G), even though these embryos failed to complete dorsal closure. As shown in Figure 4H, the JNK reporter remains active throughout tissue shrinkage (Figures 4F–4G), suggesting that, as dorsal edge cells are eliminated, adjacent epidermal cells activate JNK. Taken together, our results suggest that the dorsal open phenotype of *schnurri* mutants is a combined consequence of tissue loss by apoptosis and lack of cell migration over the amnioserosa.

Most functions of Dpp signaling are achieved through Schnurri-dependent repression of the transcriptional repressor Brinker. However, as we have shown, inhibition of *reaper* by Schnurri is direct and, hence, likely Brinker independent. By contrast, the migration of dorsal edge cells is mediated by Brinker repression, since *schnurri brinker* double mutant larvae have a sealed dorsal midline (Marty et al., 2000, Torres-Vazquez et al., 2001). It is interesting that the dorsal epidermis of these double mutants is much reduced in surface area compared to that of wild-type embryos or *brinker* single mutant embryos, which have expanded dorsal fates (in Torres-Vazquez et al., 2001, compare Figure 2D to Figures 2A and 2B). We suggest that *schnurri brinker* double mutant embryos complete dorsal closure despite tissue loss, perhaps because a sufficient number of cells are able to migrate before undergoing apoptosis. To assess directly whether Brinker has any impact on *reaper* expression, we performed gain- and loss-of-function experiments (Figures S3E–S3I). No ectopic *reaper* expression was seen in embryos lacking or overexpressing *brinker* (Figures S3G and S3H). Moreover, Brinker overexpression did not prevent *reaper* expression in *crumbs* mutant embryos (Figures S3E and S3F), while overexpressing Schnurri did (Figures S3E and S3I). We conclude that Brinker does not affect *reaper* expression and that Schnurri contributes to dorsal closure through two parallel routes: first, by repressing Brinker and, hence, allowing the derepression of genes regulating the cytoskeletal functions required for cell migration (Fernández et al., 2007, Homsy et al., 2006); and second, by repressing *reaper*, thus ensuring survival of the dorsal edge cells in the face of JNK’s proapoptotic pressure. Our results highlight the central role of Dpp and Schnurri in cell survival, extending observations on Dpp in imaginal discs (Adachi-Yamada et al., 1999, Gibson and Perrimon, 2005, Moreno et al., 2002, Shen and Dahmann, 2005) and TGFβ in vertebrates (Sabapathy et al., 1999, Taya et al., 1999).

### Conclusions

In many contexts, JNK signaling leads to apoptosis, perhaps a remnant of this pathway’s ancestral function in stress response (Ríos-Barrera and Riesgo-Escovar, 2013). JNK signaling may have started to regulate cytoskeletal functions in multicellular organisms to facilitate delamination or extrusion of defective cells. It is conceivable that such a regulatory relation might have subsequently been coopted to control other migratory behaviors such as those required for dorsal closure. Evidently, this would have necessitated coevolution of a protective, anti-apoptotic mechanism. At the dorsal edge of the epidermis, this is mediated by Dpp, which, intriguingly, is itself under the control of JNK signaling. Therefore, under the right circumstances, JNK contributes to the mechanism that counteracts its own AP-1-dependent proapoptotic pressure. The outcome of the regulatory network linking JNK and Dpp to *reaper* must be finely balanced, since a mild increase in JNK signaling (e.g., in *puckered* heterozygous embryos) triggers *reaper* expression (Kolahgar et al., 2011). Unlike the ventral epidermis, the dorsal epidermis seems prone to expressing Dpp in response to JNK, perhaps because of earlier expression in this region or through the action of additional regulators. This is likely to ensure the survival of dorsal edge cells during their migration. As we have shown, the opposing influences of Dpp and JNK are played out within the regulatory region of *reaper.* The anti-apoptotic activity of Dpp is mediated by Schnurri, a protein that could have more general anti-apoptotic activity since mammalian Schnurri has been shown to dampen cell death during T cell development (Staton et al., 2011). The function of this or similar regulatory modules in other tissues, contexts, and models can thus yield a broader understanding of the balance between apoptosis and survival at the intersection of signaling pathways.

## Experimental Procedures

Details on materials and methods can be found in the Supplemental Information. These include a full list of the *Drosophila* strains and antibodies as well as step-by-step staining protocols for immunofluorescence and in situ hybridization. For gamma irradiation, embryos were collected for 4 hr, aged a further 4 hr, and introduced in a gamma-cell irradiator for a 4,000 rad exposure (Nordstrom et al., 1996). The embryos were allowed to recover for 2 hr at 25°C before further analysis. The *reaper* reporter constructs were created by standard molecular biology with primers listed in the Supplemental Information. They were introduced into the *Drosophila* genome by PhiC31-mediated integration into PBac[yellow[+]-attP-9A] VK00027 (Bloomington *Drosophila* Stock Center # 9744, on chromosome III). EMSAs were largely performed as described elsewhere (Pyrowolakis et al., 2004). Live embryo imaging was performed either by classical confocal or MuVi-SPIM. For classical confocal microscopy, we used a Leica SP5 microscope equipped with a resonant scanner and a 20× (NA, 0.8) water immersion objective. For MuVi-SPIM, we used a custom-built set-up (two Nikon 10× 0.3-NA illumination objective lenses and two Nikon 25× 1.1-NA detection lenses) and protocols as described elsewhere (Krzic et al., 2012). The custom-modified Hamamatsu Flash 4 cameras were operated in the Lightsheet Readout Mode to reject scattered photons. Details on image processing and data visualization are provided in the Supplemental Information.

## Supplemental Information

Document S1. Supplemental Experimental Procedures and Figures S1–S4

Movie S1. JNK Activity in Wild-Type and *crumbs* Mutants, Related to Figure 1 Activity of TRE-GFP, a reporter of JNK activity, in an otherwise wild-type embryo (left) or a *crumbs* mutant embryo (right) from stage 10/11 onward. In the wild type, GFP fluorescence can be seen at the dorsal edge during dorsal closure, while in the mutant, widespread fluorescence becomes detectable as development proceeds. In this and in Movie S2, embryos are oriented with anterior on the left and dorsal at the top.

Movie S2. Dorsal Closure Fails in *schnurri* Mutants, Related to Figure 4 Dorsal closure in embryos carrying spider-GFP to mark cell outlines (lateral view showing dorsal closure at the top). Dorsal closure proceeds normally in the control embryo (left, *schnurri* heterozygote) but is impaired in the *schnurri* mutant (right). Note progressive shrinkage of the dorsal epidermis in the *schnurri* mutant during this period.

Movie S3. In Toto Imaging of Dorsal Closure in a Wild-Type and a *schnurri* Mutant, Related to Figure 4 Whole-embryo imaging (MuVi-SPIM) of a wild-type (control) followed by a *shn* mutant embryo (second half of the movie) carrying histone-RFP. Initially, eight different views, each rotated by 45°, are shown to highlight distinct viewpoints around the embryo, starting with a dorsal view (followed by lateral, ventral, lateral on subsequent views). Subsequent frames show development during and after dorsal closure. Anterior is to the center of the rosette made up by the eight views. The first half of the movie shows the wild-type (control) embryo, while the second half shows a *shn* mutant, first from the dorsal side and then every 45°. Observation over time and from several angles confirms the progressive loss of epithelial tissue from the dorsal edge, where some cell debris can be seen and where macrophages later congregate.

Movie S4. Differences between Wild-Type and *schnurri* Mutants at Key Stages of Dorsal Closure, Related to Figure 4 Control (top) and *schnurri* mutant (bottom) embryos at three representative time points (before, during, and after dorsal closure). Each embryo undergoes a 360° rotation around the anterior-posterior axis to allow viewing of the whole epidermis (generated from MuVi-SPIM data). This shows the progressive loss of epidermal cells in the mutant, while dorsal cells migrate normally over the amnioserosa in the wild-type.

Document S2. Article plus Supplemental Information
