# Supplementary material for: The Dpp/TGFβ-Dependent Corepressor Schnurri Protects Epithelial Cells from JNK-Induced Apoptosis in Drosophila Embryos
Source: Dev Cell. 2014 Oct 27;31(2):240–7. doi: 10.1016/j.devcel.2014.08.015 (PMC4220000; doi:10.1016/j.devcel.2014.08.015)
Supplement: Document S1. Supplemental Experimental Procedures and Figures S1–S4 [file mmc1.pdf]

Developmental Cell, Volume *31*
Supplemental Information

# The Dpp/TGFβ-Dependent Corepressor Schnurri Protects Epithelial Cells from JNK-Induced Apoptosis in *Drosophila* Embryos

Jorge V. Beira, Alexander Springhorn, Stefan Gunther, Lars Hufnagel, Giorgos Pyrowolakis, and Jean-Paul Vincent

**Figure S1**

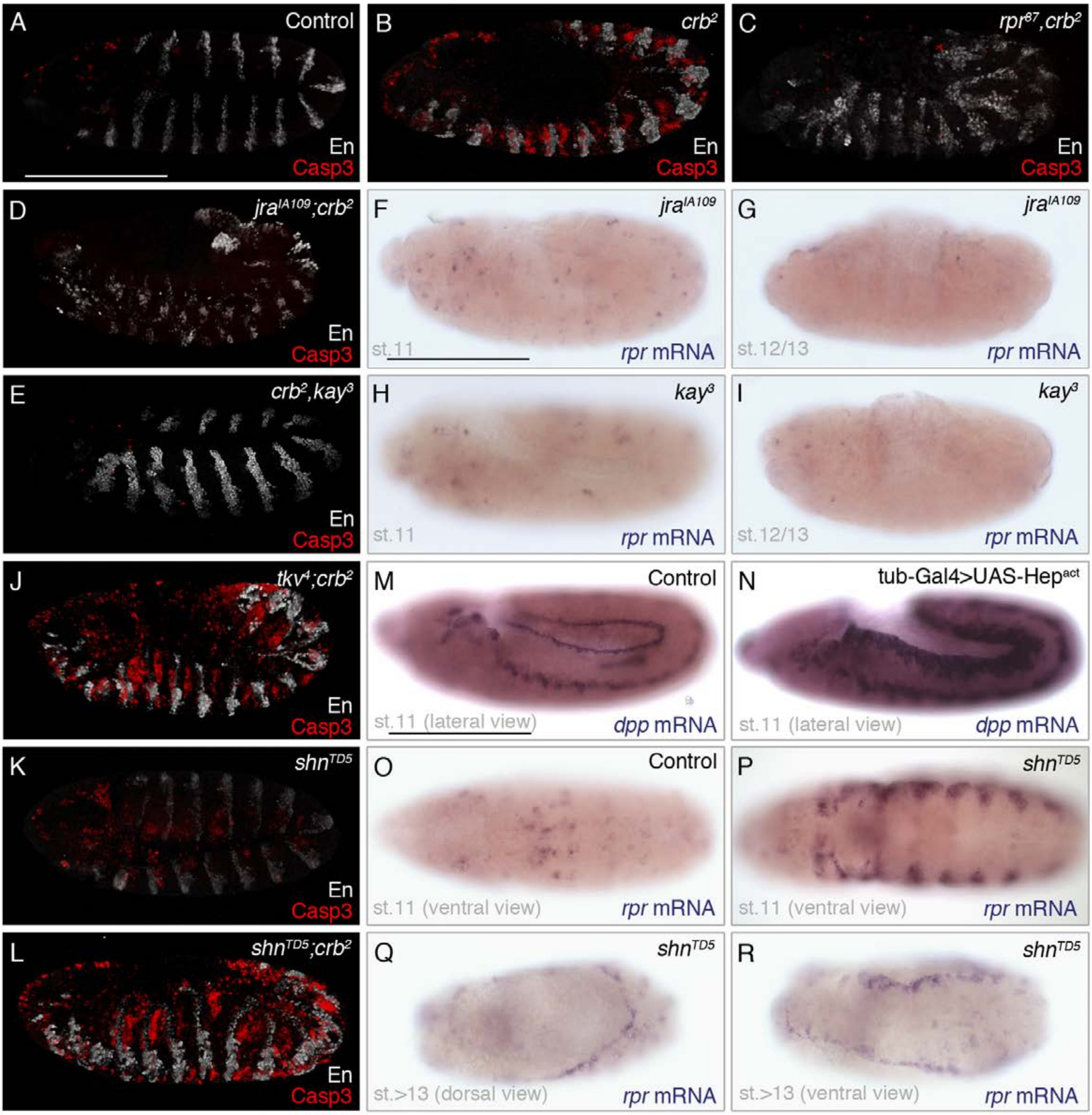

A Control
En
Casp3
B crb2
En
Casp3
C rpr87,crb2
En
Casp3
D jraIA109;crb2
En
Casp3
F jraIA109
st.11
rpr mRNA
G jraIA109
st.12/13
rpr mRNA
E crb2,kay3
En
Casp3
H kay3
st.11
rpr mRNA
I kay3
st.12/13
rpr mRNA
J tkv4;crb2
En
Casp3
M Control
st.11 (lateral view)
dpp mRNA
N tub-Gal4>UAS-Hepact
st.11 (lateral view)
dpp mRNA
K shnTD5
En
Casp3
O Control
st.11 (ventral view)
rpr mRNA
P shnTD5
st.11 (ventral view)
rpr mRNA
L shnTD5;crb2
En
Casp3
Q shnTD5
st.>13 (dorsal view)
rpr mRNA
R shnTD5
st.>13 (ventral view)
rpr mRNA

## SUPPLEMENTAL FIGURES AND LEGENDS

**Figure S1 (related to Figure 1). The apoptotic response to epithelial disruption is mediated by *reaper* and modulated by Schnurri. (A-B)** Occasional apoptotic cells, marked with anti-Caspase-3, can be seen in control embryos (A) while apoptosis is substantially increased in the ventral epidermis of *crumbs* mutants (B). (**C**) Anti-Caspase-3 staining shows that only a small number of apoptotic cells appear in *rpr*$^{87}$*crumbs* double mutants. (**D-E**) Staining of double mutants also show that *jun (jra)* and *fos (kayak)* are required for apoptosis in *crumbs* mutants. (**F-I**) Absence of *reaper* expression in *jun* (*jra*) or *fos* (*kay*) single mutants, as seen in *jun crumbs* and *crumbs kayak* double mutants shown in Fig. 1. (**J-L**) Widespread apoptosis in the epidermis of *tkv crumbs* double mutants (**J**). Ectopic apoptosis in the dorsal epidermis of *shn* mutants (**K**). Caspase3 immunoreactiviy is seen throughout the epidermis of *shn crumbs* double mutants (**L**), including in the dorsal epidermis, which is protected in *crumbs* single mutants. (**M**) Expression of *dpp* in a control embryo (*tubulin-gal4/*+). (**N**) Over-expression of Hemipterous, Drosophila JNKK (*tubulin-gal4/+, UAS-Hep/*+), leads to excess transcription of *dpp*. (**O-P**) Expression of *reaper* is upregulated in the dorso-lateral epidermis of stage 11 *schnurri* mutant embryos (P). Compare to control embryo (O, *shn[TD5]/+)*. Ventral views are used to show the lateral extent of expression. (**Q-R**) At later stages (>13) a band of expression remains at the dorsal edge of the epidermis. Dorsal view at this stage shows the gaping hole (R), while ventral view shows all the epidermis that remains.

**Figure S2**

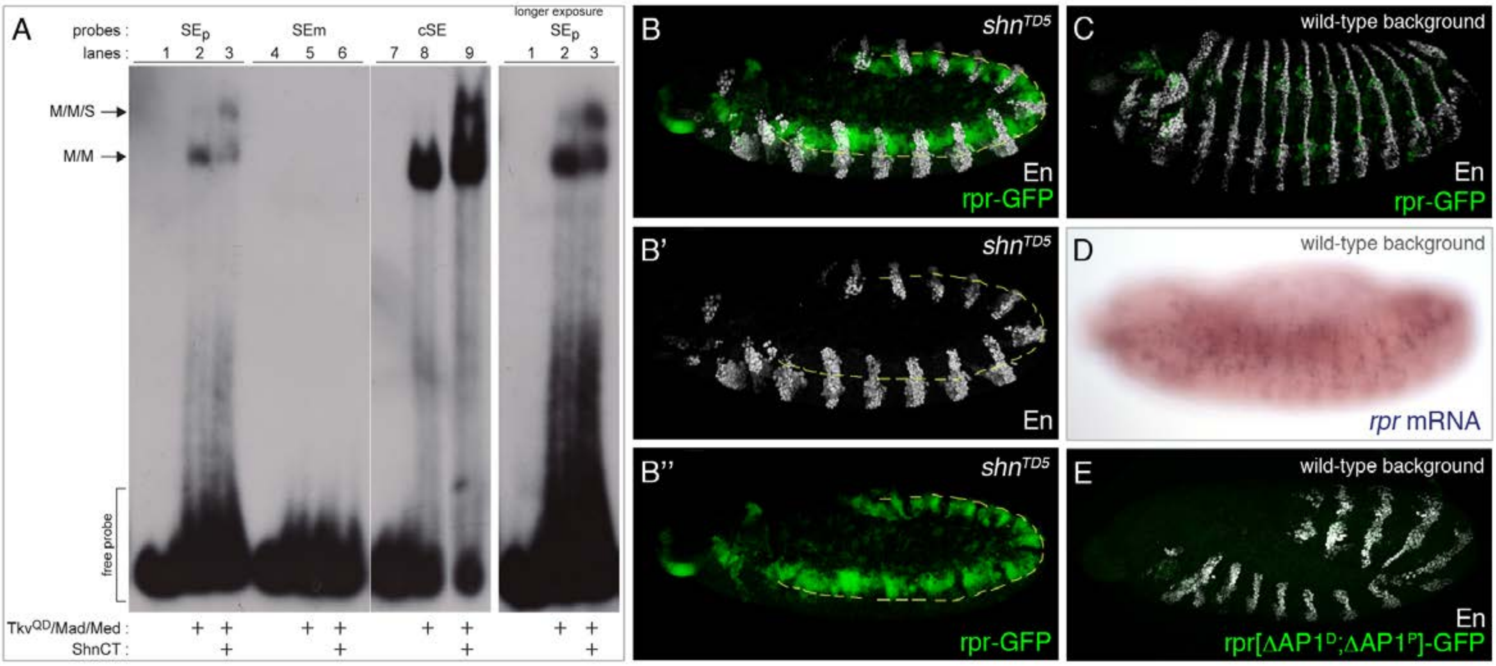

A
probes :
SEp
SEm
cSE
longer exposure
SEp
lanes :
1 2 3
4 5 6
7 8 9
1 2 3
M/M/S
M/M
free probe
TkvQD/Mad/Med :
ShnCT :
B
shnTD5
En
rpr-GFP
B'
shnTD5
En
B''
shnTD5
rpr-GFP
C
wild-type background
En
rpr-GFP
D
wild-type background
rpr mRNA
E
wild-type background
En
rpr[ΔAP1D;ΔAP1P]-GFP

**Figure S2 (related to Figure 2). JNK and Dpp signaling regulate *reaper* expression directly on the promoter.** (**A**) Binding of the Schnurri/Mad/Medea complex as detected by EMSA, on the proximal silencer element ($SE_p$) from the *reaper* promoter (referred to as $SE_p$ in the text). No binding can be detected on the mutated element (SEm; same mutation as in the *rpr[ΔShn]-GFP* transgenic reporter). The previously characterized SE from *brinker* is used as a positive control (cSE). The indicated probes were incubated with extracts from S2 cells transfected with constitutively active Thickveins (TkvQD), Mad and Medea (to generate pMad/Med complexes (M/M)) and with an N-terminally truncated version of Schnurri (ShnCT) (to generate pMad/Medea/ Schnurri complexes (M/M/S)). (**B**) Expression of the *rpr-GFP* reporter in *schnurri* mutants (like in Fig. 3D) is segmentally modulated; separate channels for GFP (B'') and Engrailed (B') are shown. (**C-D**) At later stages (stage 13, 14 and later), *rpr-GFP* has a segmental expression in ventral groups of cells of wild-type embryos (C), similarly to endogenous *reaper* at these later stages (D). (**E**) Weak expression of *rpr[ΔAP1$^P$;ΔAP1$^D$]-GFP* in an otherwise wild-type background (compare to Fig. 2J and 2B). As mentioned in the main text, expression of *rpr[ΔShn]-GFP* in wild type embryos is delayed relative to that of *rpr-GFP* in *schnurri* mutants. Timely activation of *reaper* expression in the dorsal epidermis could conceivably require an enhancer that is missing in the reporter. Alternatively, it is possible that loss of Schnurri not only removes *reaper* repressor activity but may also, in addition, indirectly allow increased JNK activity in the dorsal epidermis, thus accounting for early activation of *rpr-GFP*. It is also worth noting the segmental expression of *rpr[ΔShn]-GFP* at stage 13 (in addition to the expected dorsal expression). This may not be directly attributable to loss of Schnurri since, at this late stage, similar though weaker expression was also detectable from the *rpr-GFP* transgene as well as from the endogenous *reaper* gene in wild type embryos (Fig. S2C,D).

**Figure S3**

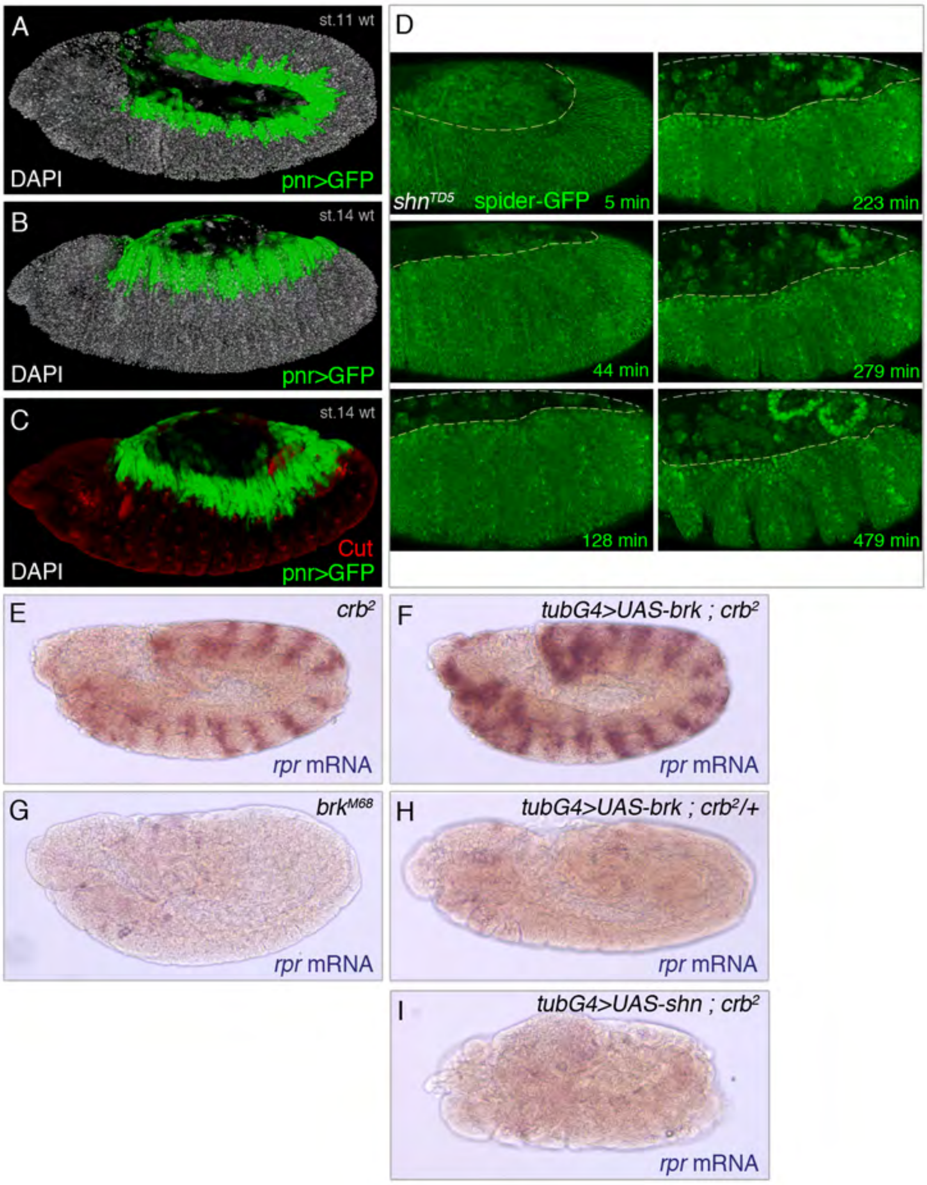
A
st.11 wt
DAPI
pnr>GFP
B
st.14 wt
DAPI
pnr>GFP
C
st.14 wt
Cut
DAPI
pnr>GFP
D
shn^TD5 spider-GFP 5 min
223 min
44 min
279 min
128 min
479 min
E
crb^2
rpr mRNA
F
tubG4>UAS-brk ; crb^2
rpr mRNA
G
brk^M68
rpr mRNA
H
tubG4>UAS-brk ; crb^2/+
rpr mRNA
I
tubG4>UAS-shn ; crb^2
rpr mRNA

**Figure S3 (related to Figure 3). The dorsal epidermis is reduced in the absence of Schnurri.** (**A-B**) Expression of *pannier* (as indicated in *pannier-gal4 UAS-GFP*), a gene activated by Dpp signaling (Winick et al., 1993) marks the dorsal epidermis, where Dpp signaling is active. (**C**) Position of the dorsal and ventral clusters of Cut expression (highlighted with anti-Cut) relative to the pannier domain (see Fig. 4). (**D**) Stills from a confocal movie of a *schnurri* mutant embryo expressing spider-GFP as a marker of cell outlines. Over time, epidermal cells appear to be lost from the dorsal side, progressively exposing the gut. Many macrophages are seen to scurry around the dorsal edge (see movie S2). (**E-H**) Expression of *reaper* following alterations in *brinker* and *schnurri* activity. *brinker* loss of function does not result in ectopic *reaper* (G). Brinker overexpression did not prevent *reaper* upregulation in *crumbs* mutants (F), while overexpression of Schnurri did (I). Moreover, Brinker overexpression had no detectable effect on *reaper* expression in control embryos (H).

**Figure S4**

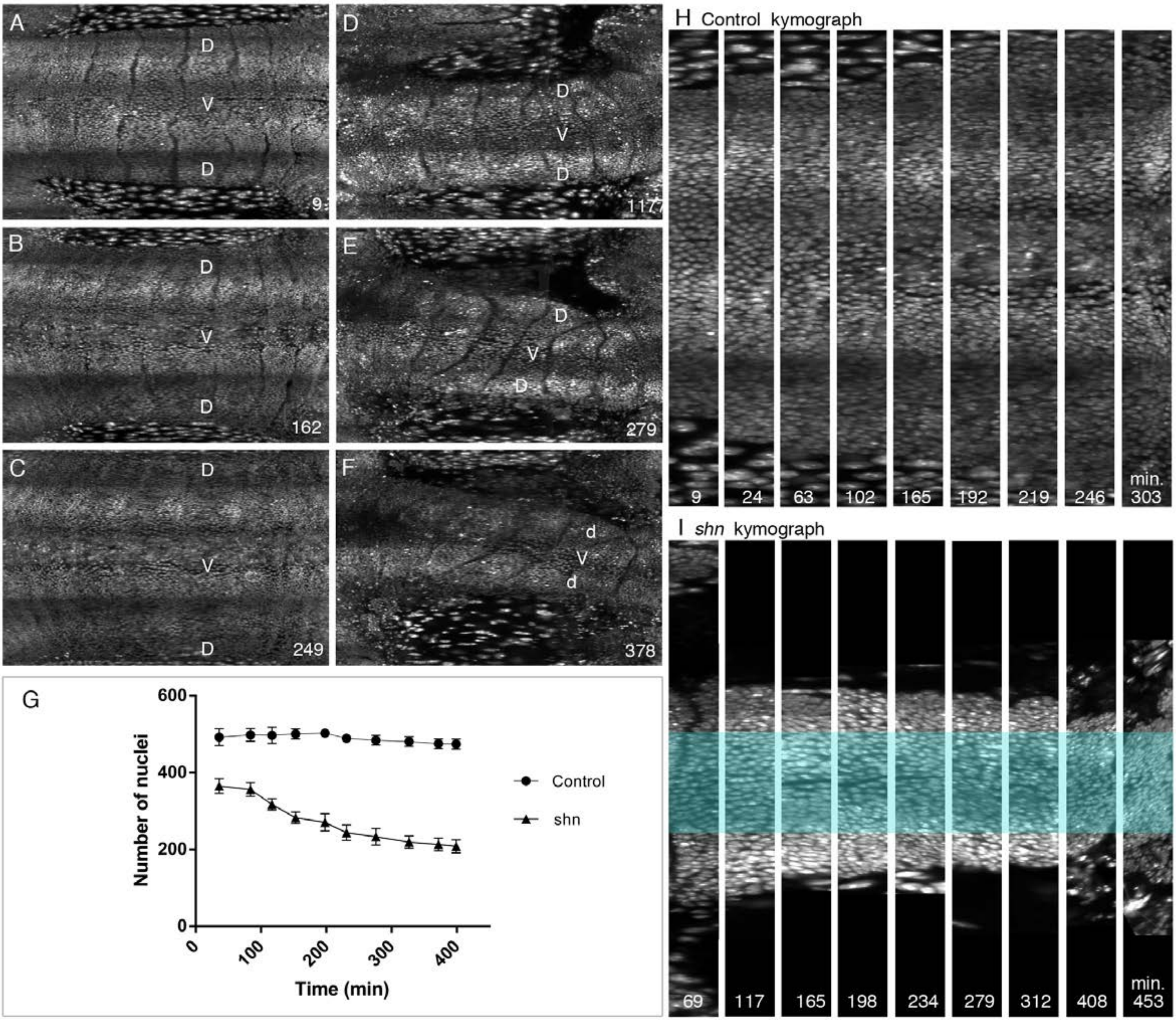
A
D
V
D
9
B
D
V
D
162
C
D
V
D
249
D
D
V
D
117
E
D
V
D
279
F
d
V
d
378
G
600
400
200
0
Number of nuclei
0
100
200
300
400
Time (min)
Control
shn
H Control kymograph
9
24
63
102
165
192
219
246
min.
303
I shn kymograph
69
117
165
198
234
279
312
408
min.
453

**Figure S4 (related to Figure 4). Dorsal closure defects in the absence of Schnurri. (A-I)** *In toto* live imaging of control and *schnurri* mutants using MuVi-SPIM. The epidermis was computationally projected on a single plane (see Supplemental Materials and Methods below) and is displayed with the ventral midline (V) horizontally in the middle. (**A-C**) In wild type embryos, migration of epidermal cells progressively covers the amnioserosa recognizable by its large nuclei (top and bottom of each frame). By contrast, dorsal closure fails in *schnurri* mutants (amnioserosa remains uncovered; **D-F**), and the dorsal epidermis (D) becomes reduced (see also movies S3 and S4). (**G**) The number of histone-RFP nuclei per segment (counted in the three central segments (7-9)) decreased over time in *schnurri* mutants, while remaining mostly constant in control embryos. (**H-I**) Such reduction is also apparent on a kymograph displaying segment 8 at selected time points. Cell debris are also seen around the dorsal edge (see also movies S3 and S4), as expected from increased apoptosis there.

**SUPPLEMENTAL MOVIE LEGENDS**

**Movie S1 (related to Figure 1).** Activity of TRE-GFP, a reporter of JNK activity, in an otherwise wild type embryo (left) or a *crumbs* mutant embryo (right) from stage 10/11 onwards. In the wild type, GFP fluorescence can be seen at the dorsal edge during dorsal closure while in the mutant widespread fluorescence becomes detectable as development proceeds. In this and in movie S2, embryos are oriented with anterior on the left and dorsal at the top.

**Movie S2 (related to Figure 4).** Dorsal closure in embryos carrying spider-GFP to mark cell outlines (lateral view showing dorsal closure at the top). Dorsal closure proceeds normally in the control embryo (left, *schnurri* heterozygote) but is impaired in the *schnurri* mutant (on the right). Note progressive shrinkage of the dorsal epidermis in the *schnurri* mutant during this period.

**Movie S3 (related to Figure 4).** Whole-embryo imaging (MuVi-SPIM) of a wild-type (control) followed by a *shn* mutant embryo (second half of the movie) carrying histone-RFP. Initially, eight different views, each rotated by 45 degrees, are shown to highlight distinct view points around the embryo, starting with a dorsal view (followed by lateral, ventral, lateral on subsequent views). Subsequent frames show development during and after dorsal closure. Anterior is to the centre of the rosette made up by the eight views. The first half of the movie shows the wild-type (control) embryo, while the second half shows a *shn* mutant, first from the dorsal side and then every 45 degrees. Observation over time and from several angles confirms the progressive loss of epithelial tissue from the dorsal edge, where some cell debris can be seen and where macrophages later congregate.

**Movie S4 (related to Figure 4).** Control (top) and *schnurri* mutant (bottom) embryos at three representative time points (before, during and after dorsal closure). Each embryo undergoes a

360 degrees rotation around the A-P axis to allow viewing of the whole epidermis (generated from MuVi-SPIM data). This shows the progressive loss of epidermal cells in the mutant, while dorsal cells migrate normally over the amnioserosa in the wild-type.

## SUPPLEMENTAL MATERIALS AND METHODS

*Drosophila genetics.* Most alleles used on this study were balanced over fluorescently marked balancer chromosomes in order to distinguish homozygous mutant embryos from their heterozygous counterparts, which were used as controls. Balancer chromosomes used include: CTG (CyO, Twist-Gal4>UAS-GFP), CKG (CyO, Krüppel-Gal4>UAS-GFP), TTG (TM3, Sb, Ser, Twist-Gal4>UAS-GFP), TDY (TM6, Hu, Sb, Deformed-YFP) and TKG (TM3, Sb, Krüppel-Gal4>UAS-GFP). The following fly stocks were used: *crb*$^{2}$*/TM6* (BDSC #3448). *crb*$^{2}$*/TTG. w;;tub-Gal4,crb*$^{2}$*/TM3. UAS-CD8-GFP, crb*$^{2}$*/TDY. Def(3L)XR38/TDY. shn*$^{TD5}$*/Cyo-wgLacZ. shn*$^{TD5}$*/CTG. TRE-GFP;crb*$^{2}$*/TDY,* JNK signaling reporter from (Chatterjee and Bohmann, 2012). *jra*$^{IA109}$*/CTG* (BDSC #3273). *kay*$^{3}$*/TTG* (from Henri Jasper, (Biteau and Jasper, 2011)). *jra*$^{IA109}$*/CKG;crb*$^{2}$*/TTG. kay*$^{3}$*,crb*$^{2}$*/TTG. rpr*$^{87}$*/TM2* (Tan et al., 2011). *rpr*$^{87}$*,crb*$^{2}$*/TTG. shn*$^{TD5}$*/CKG;crb*$^{2}$*/TTG. shn*$^{TD5}$*/CKG;kay*$^{3}$*/TTG. tub-Gal4/TKG. UAS-Hep*$^{act}$ (from Dirk Bohmann). *UAS-dppRNAi* (BDSC #36779) (Ni et al., 2011). *tkv*$^{4}$*/CTG. tkv*$^{4}$*/CKG;crb*$^{2}$*/TTG. shn*$^{TD5}$*/CKG;spider-GFP. shn*$^{TD5}$*/CyO; histone-GFP* (or his-RFP). *rpr-GFP. shn*$^{TD5}$*/CKG;rpr-GFP. shn*$^{TD5}$*,TRE-DsRed/CKG;rpr-GFP. rpr-GFP,crb*$^{2}$*/TTG. shn*$^{TD5}$*/CKG;rpr-GFP,crb*$^{2}$*/TTG. rpr[ΔShn]-GFP. rpr[ΔShn]-GFP,crb*$^{2}$*/TTG. rpr[ΔAP1*$^{P}$*]-GFP. rpr[ΔAP1*$^{P}$*]-GFP*;*crb*$^{2}$*/TTG. rpr[AP1*$^{D}$*]-GFP. rpr[ΔAP1*$^{D}$*]-GFP*;*crb*$^{2}$*/TTG. rpr[ΔAP1*$^{P}$*;ΔAP1*$^{D}$*]-GFP. rpr[ΔAP1*$^{P}$*;ΔAP1*$^{D}$*]-GFP*;*crb*$^{2}$*/TTG.*

*Immunofluorescence and in situ hybridization.* Embryos were fixed in 6% formaldehyde/heptane interface for 20 min and then devitellinised with methanol. They were

stored in methanol until further processing. In situ hybridisation to a digoxigenin-labelled single stranded RNA probe was performed at 63°C without proteinase K (Kolahgar et al., 2011). When necessary, hybridized embryos were stained with anti-GFP to confirm the genotype. Hybridized embryos were mounted in 1:1 glycerol / PBS photographed with a Zeiss Axiophot-2 microscope coupled to a Zeiss AxioCam HRC camera and the Axiovision Rel. 4.7 software. Immunofluorescence was performed according to standard protocols. The following primary antibodies were used: mouse anti-Engrailed (mAb4D9, Developmental Studies Hybridoma Bank (DSHB), 1/200), mouse anti-Wingless (mAb4D4, DSHB, 1/1000), mouse anti-p-Smad (mAb pS423/425, Epitomics, 1/200), chicken anti-GFP (Abcam, 1/1000), and chicken anti-β-Galactosidase (Abcam cat 13970-100; 1/1000). Appropriate secondary antibodies were obtained from Molecular Probes. Stained embryos were mounted in Vectashield (Vector laboratories) or glycerol 50% diluted in PBS and stored at 4 °C. Images were acquired using a Leica SP5 laser-scanning confocal microscope and assembled in ImageJ/Fiji (NIH), Photoshop (Adobe) or Volocity (Perkin Elmer).

*Reporter constructs.* The upstream region of *reaper* including the transcription start site (5.5 kb) was amplified from genomic DNA by PCR with the following primers: 5'-CACCCGGAAAATGGAAATGTAAG-3' (forward) and 5'-ATGATTTTTTTCGAGATGCG-3' (reverse) and cloned in front of a GFP cDNA. This 'wild type' reporter (*rpr-GFP*) was then used as a template for directed mutagenesis to generate the variant reporters described in the text. All the reporters were introduced into the *Drosophila* genome by PhiC31-mediated integration into PBac[yellow[+]-attP-9A] VK00027 (BDSC# 9744, on Chr. III). The transgenes were also inserted into PBac[yellow[+]-attP-3B] VK00002 (BDSC# 9723, on Chr. II), which gave similar (not shown) results.

*EMSA*. For electrophoretic mobility shift assays (EMSA), *Drosophila* S2 cells were either transfected with expression plasmids for $Tkv^{QD}$ (50 ng), Mad (175 ng) and Med (175 ng) or with expression plasmids for ShnCT (400 ng). Cells were harvested 72 hr after transfection and lysed for 10 min at 4°C in 100 µl of 100 mM Tris (pH 7.5), 1 mM DTT, 0.5% TritonX100 and 1% NP40. Radioactively labeled probes were generated by annealing and filling in partially overlapping oligonucleotides in the presence of α-32 dATP. Binding reactions were carried out in a total volume of 25 µl containing 12.5 µl 2x binding buffer (200 mM KCl, 40 mM HEPES (pH 7.9), 40% glycerol, 2 mM DTT, 0.6% BSA and 0.02% NP40), 10000 cpm of radioactively labeled probe, 1 µl poly dIdC (1mM) and 7 µl of cleared S2 cell extracts. After incubation for 30 min at 4°C, the reactions were analyzed by non-denaturing 4% polyacrylamide gel electrophoresis followed by autoradiography (Pyrowolakis et al., 2004).

*Live imaging by classical confocal microscopy.* After dechorionation in 50% bleach for 2 minutes, embryos were washed with water. Live embryos of the appropriate stage and genotype were selected under a fluorescence microscope. They were then mounted on a thin strip of glue at the bottom of a Petri dish, covered with PBS, and imaged at 22°C in the Live Data mode of an upright Leica SP5 microscope equipped with a resonant scanner. A 20x (NA=0.8) water immersion objective (Leica) was used. Typical acquisition time was 40-55 seconds/embryo. The time-lapse series were assembled and analysed with ImageJ/Fiji.

*Live imaging by MuVi-SPIM.* Embryos were imaged with a custom-built MuVi-SPIM setup with two illumination objective lenses (Nikon 10x 0.3NA) and two detection lenses (Nikon 25x 1.1NA), as previously described (Krzic et al., 2012). Two custom-modified Hamamatsu Flash 4 cameras were used to record the opposing views of the sample. These cameras were operated in the Lightsheet Readout Mode™ to reject scattered photons (De Medeiros et al., 2014). Embryo

preparation and mounting in transparent gels was performed as previously described (Krzic et al., 2012). Every three minutes, two perpendicular oriented z-stacks (each with 200 planes and 1 μm spacing) covering the entire embryo were recorded. These stacks were computationally fused into a single 3D dataset for each time point. Slight shifts of the embryo in the gel over the 14hrs image period were compensated by a generic particle image velocimetry method written in Matlab (www.mathworks.com). Epidermis projections: for *in toto* analysis and visualization of development of the embryonic epidermis, the dimensionality of the entire dataset (3D images and time) was reduced by projecting the surface of the embryo to a plane; first, for each time point, we determined the convex hull of the 3D surface of the embryo based on the fluorescent intensity distribution in the 3D image. A standard Mercator projection was then applied to map the embryonic surface to a plane. Despite this projection having an effect on size and shape of objects at the embryo poles, the analysis was done in the central locations where it yields an unaltered representation of the tissue away from the poles. A multi-slice Mercator projection was generated, covering a thin layer around the embryos surface using Python (www.python.org). A secondary maximum projection of the multiple Mercator slices into a single 2D image provides a complete view of the fluorescent intensity distribution of the embryos surface, thus the epidermis. For the movies, volumetric rendering of the 3D fluorescent intensity distributions were performed using Fiji (www.fiji.sc) with the 3D-Viewer plugin. The rendering yields a 2D view of the fluorescence distribution as observed from a viewpoint (e.g. dorsal and ventral) outside of the embryo. Viewpoints and time points were rendered independently and then stitched together.

## SUPPLEMENTAL REFERENCES


Biteau, B., and Jasper, H. (2011). EGF signaling regulates the proliferation of intestinal stem cells in Drosophila. Development *138*, 1045-1055.

Chatterjee, N., and Bohmann, D. (2012). A versatile ΦC31 based reporter system for measuring AP-1 and Nrf2 signaling in Drosophila and in tissue culture. PLoS ONE *7*, e34063.

De Medeiros, G.Q.G., Norlin, N., Gunther, S., Albert, M., Krzic, U., and Hufnagel, L. (2014). Multiview light-sheet microscopy goes confocal. *Under review*.

Kolahgar, G., Bardet, P.-L., Langton, P.F., Alexandre, C., and Vincent, J.-P. (2011). Apical deficiency triggers JNK-dependent apoptosis in the embryonic epidermis of Drosophila. Development *138*, 3021-3031.

Krzic, U., Gunther, S., Saunders, T.E., Streichan, S.J., and Hufnagel, L. (2012). Multiview light-sheet microscope for rapid in toto imaging. Nature methods *9*, 730-733.

Ni, J.-Q., Zhou, R., Czech, B., Liu, L.-P., Holderbaum, L., Yang-Zhou, D., Shim, H.-S., Tao, R., Handler, D., Karpowicz, P*., et al.* (2011). A genome-scale shRNA resource for transgenic RNAi in Drosophila. Nature methods *8*, 405-407.

Pyrowolakis, G., Hartmann, B., Müller, B., Basler, K., and Affolter, M. (2004). A simple molecular complex mediates widespread BMP-induced repression during Drosophila development. Developmental cell *7*, 229-240.

Tan, Y., Yamada-Mabuchi, M., Arya, R., St Pierre, S., Tang, W., Tosa, M., Brachmann, C., and White, K. (2011). Coordinated expression of cell death genes regulates neuroblast apoptosis. Development *138*, 2197-2206.

Winick, J., Abel, T., Leonard, M.W., Michelson, A.M., Chardon-Loriaux, I., Holmgren, R.A., Maniatis, T., and Engel, J.D. (1993). A GATA family transcription factor is expressed along the embryonic dorsoventral axis in Drosophila melanogaster. Development *119*, 1055-1065.
