# Supplementary material for: The Dpp/TGFβ-Dependent Corepressor Schnurri Protects Epithelial Cells from JNK-Induced Apoptosis in Drosophila Embryos
Source: Dev Cell. 2014 Oct 27;31(2):240–7. doi: 10.1016/j.devcel.2014.08.015 (PMC4220000; doi:10.1016/j.devcel.2014.08.015)
Supplement: Document S2. Article plus Supplemental Information [file mmc6.pdf]

Developmental Cell
Short Article

The Dpp/TGFβ-Dependent Corepressor Schnurri Protects Epithelial Cells from JNK-Induced Apoptosis in *Drosophila* Embryos

Jorge V. Beira,[1,2,*] Alexander Springhorn,[3,4] Stefan Gunther,[5] Lars Hufnagel,[5] Giorgos Pyrowolakis,[3] and Jean-Paul Vincent[1,*]
[1]Medical Research Council National Institute for Medical Research, The Ridgeway, Mill Hill, London NW7 1AA, UK
[2]Department of Cell and Developmental Biology, University College London, Gower Street, London WC1E 6BT, UK
[3]BIOSS Centre for Biological Signalling Studies and Institute for Biology I, University of Freiburg, Schänzlestrasse 18, 79104 Freiburg, Germany
[4]Spemann Graduate School of Biology and Medicine, Research Training Program GRK 1104, Albert Ludwigs University, 79104 Freiburg, Germany
[5]European Molecular Biology Laboratory, Meyerhofstrasse 1, 69117 Heidelberg, Germany
*Correspondence: jbeira@gmail.com (J.V.B.), jvincen@nimr.mrc.ac.uk (J.-P.V.)
http://dx.doi.org/10.1016/j.devcel.2014.08.015
This is an open access article under the CC BY license (http://creativecommons.org/licenses/by/3.0/).

SUMMARY

Jun N-terminal kinase (JNK) often mediates apoptosis in response to cellular stress. However, during normal development, JNK signaling controls a variety of live cell behaviors, such as during dorsal closure in *Drosophila* embryos. During this process, the latent proapoptotic activity of JNK becomes apparent following Dpp signaling suppression, which leads to JNK-dependent transcriptional activation of the proapoptotic gene *reaper.* Dpp signaling also protects cells from JNK-dependent apoptosis caused by epithelial disruption. We find that repression of *reaper* transcription by Dpp is mediated by Schnurri. Moreover, reporter gene analysis shows that a transcriptional regulatory module comprising AP-1 and Schnurri binding sites located upstream of *reaper* integrate the activities of JNK and Dpp. This arrangement allows JNK to control a migratory behavior without triggering apoptosis. Dpp plays a dual role during dorsal closure. It cooperates with JNK in stimulating cell migration and also prevents JNK from inducing apoptosis.

INTRODUCTION

Signaling by c-Jun N-terminal kinase (JNK) mediates one of the major stress response pathways (Chen, 2012; Stronach and Perrimon, 1999). Indeed, activation of JNK signaling often boosts or triggers apoptosis (Dhanasekaran and Reddy, 2008; Igaki, 2009; Leppä and Bohmann, 1999). JNK can exert its proapoptotic effect through phosphorylation of Jun, a component of the AP-1 transcriptional activator, or of other cellular proteins (Bogoyevitch and Kobe, 2006). It is important to note, however, that JNK signaling does not always trigger apoptosis (Weston and Davis, 2007) and has been shown to control nonapoptotic processes such as cytoskeletal rearrangements (Homsy et al., 2006), cell migration (Ríos-Barrera and Riesgo-Escovar, 2013), and cell proliferation (Shaulian and Karin, 2002) during development and regeneration. It is generally thought that the cellular context or the activity of other signaling pathways determines whether JNK signaling leads to apoptosis or not. In well-documented instances, this involves downregulation or blunting of JNK signaling itself, e.g., through the activity of Gadd45β, an NF-κB-induced factor (De Smaele et al., 2001; Papa et al., 2004), or by Puckered, a feedback inhibitor of JNK signaling (McEwen and Peifer, 2005). Mechanisms that dampen JNK's proapoptotic influence without affecting core pathway activity have also been documented. For example, in the developing *Drosophila* eye, mitogen-activated protein kinase phosphorylates and destabilizes Hid, a proapoptotic protein transcriptionally activated by JNK signaling in this tissue (Bergmann et al., 1998). Another documented process involves transcriptional repression of *hid*, which would otherwise be overactivated by JNK in response to irradiation damage (Luo et al., 2007). Non-cell-autonomous protective mechanisms could also be at work. For example, the transcriptional modulator Schnurri limits radiation-induced tissue damage by recruiting macrophages through activation of the PDGF-related growth factor Pvf1 (Kelsey et al., 2012). All the aforementioned mechanisms have been shown to operate in response to cellular stress. However, so far, little is known about the regulatory processes that prevent JNK from causing apoptosis during normal development. One well-characterized nonapoptotic JNK-dependent developmental process is the morphogenetic movement of dorsal closure (Glise and Noselli, 1997; Hou et al., 1997; Riesgo-Escovar and Hafen, 1997). Here, we set out to investigate the molecular mechanisms that prevent JNK from activating apoptosis during this process.

Dorsal closure is a morphogenetic movement that closes a large gap left on the embryo's dorsal side after germ band retraction. It involves the concerted movement of the dorsal epidermis toward the midline and requires both JNK and Dpp signaling. One current view is that JNK signaling at the leading edge promotes expression of Decapentaplegic (Dpp, a transforming growth factor β [TGF-β] homolog), which, in turn, orchestrates

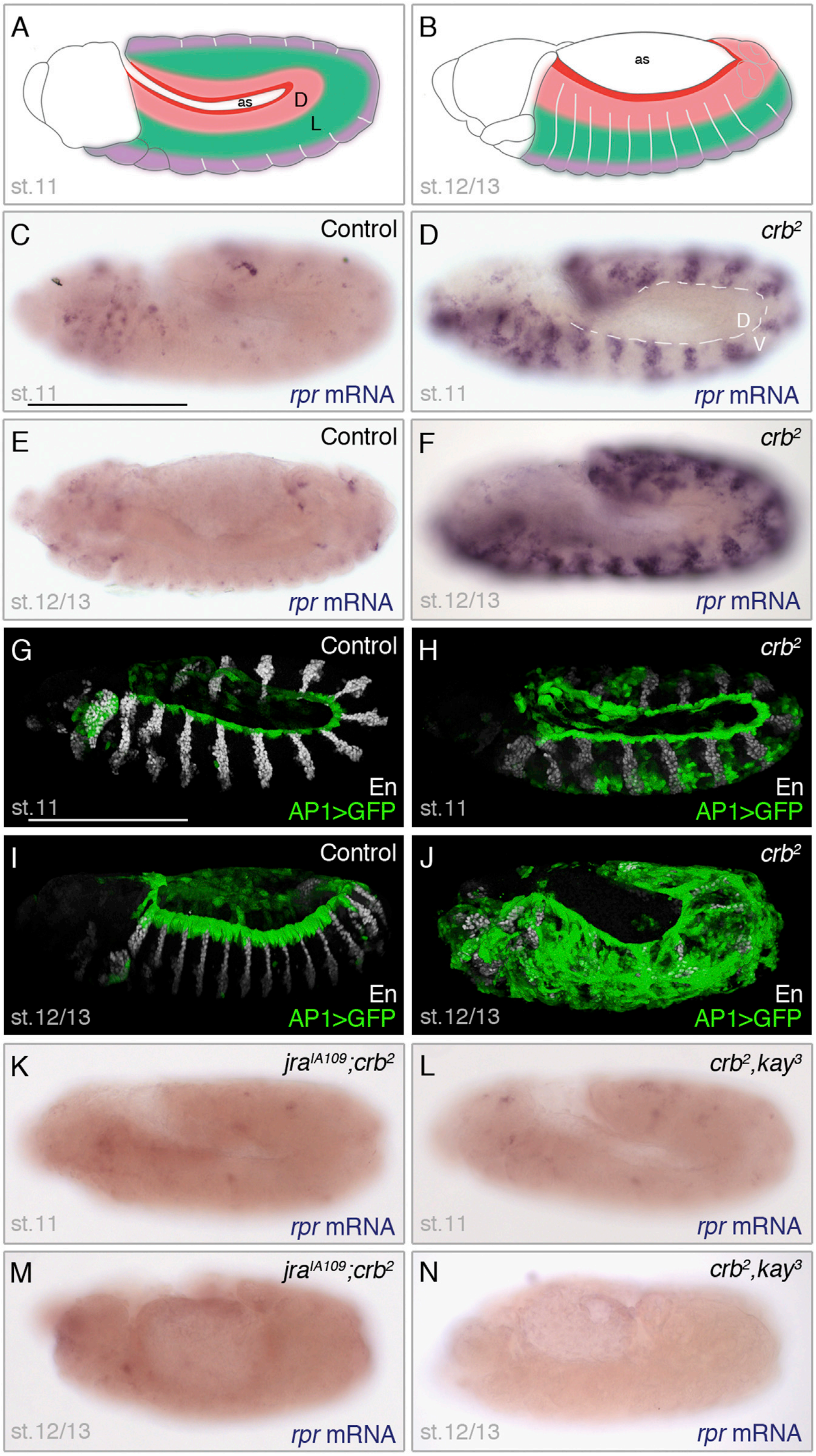

Figure 1. Epithelial Disruption Triggers Canonical JNK Signaling, which, in Turn, Activates *reaper* Transcription in the Ventral and Lateral Epidermis of *Drosophila* Embryos

(A and B) Diagram of *Drosophila* embryos at stage (st.) 11 and stage 12/13, indicating the key epidermal domains: the dorsal (D) edge (deep red), the dorsal epidermis (faded red), the lateral (L) epidermis (green), and the ventral epidermis (purple). as, amnioserosa.

(C–F) Expression of *reaper* in control (*crb*^{2}/+) and *crumbs* mutant (*crb*^{2}/ *crb*^{2}) embryos at stages 11 (C and D) and 12/13 (E and F), as indicated. In homozygous *crumbs* mutants, segmental upregulation is seen in the ventrolateral (V) epidermis, while in the dorsal (D) epidermis (inside white dotted lines), it remains silent. mRNA, messenger RNA.

(G–J) A fluorescent reporter of JNK activity (Chatterjee and Bohmann, 2012) is active at the edge of the dorsal epidermis of control embryos (*crb*^{2}/+) and throughout the epidermis of homozygous *crumbs* mutant embryos (*crb*^{2}/ *crb*^{2}). Engrailed (En) immunoreactivity provides positional landmarks along the anterior-posterior axis. Embryonic stages are indicated.

(K–N) Expression of *reaper* is not activated in *crumbs* mutant embryos that also lack *jra* (jun) or *kayak* (fos). As positive controls, *crumbs* mutant embryos were stained in parallel (data not shown). Genotypes and stages are indicated.

Scale bar, 200 μm.

the cell shape changes required for dorsal closure (Fernández et al., 2007; Ríos-Barrera and Riesgo-Escovar, 2013). It is likely that a protective mechanism is at work at the leading edge since JNK does not trigger apoptosis there. Additional evidence that the dorsal epidermis is protected from apoptosis came from the analysis of *crumbs* (*crb*) (abbreviated as *crb* in genotypic descriptions) mutants, where JNK target genes are upregulated in response to loss of apicobasal polarity (Kolahgar et al., 2011). In such embryos, most epidermal cells undergo apoptosis, except in an approximately ten-cell-wide band of dorsal cells, despite strong activation of JNK signaling there. It appears, therefore, that the protective mechanism acts over a broader domain than just within the leading edge (see Figures 1A and 1B for a diagram of the relevant region of the embryonic epidermis).

Most apoptosis in *Drosophila* requires the *H99* locus (White et al., 1994), which comprises the three main proapoptotic genes: *reaper*, *hid*, and *grim*. Among these, *reaper* is the most likely mediator of the response to epithelial disruption since it is upregulated in *crb* mutant embryos in a pattern prefiguring that of caspase immunoreactivity (Kolahgar et al., 2011). Moreover, overexpression of Puckered, a phosphatase that inhibits JNK signaling prevents *reaper* upregulation, as well as apoptosis, in *crb* mutants (Kolahgar et al., 2011).

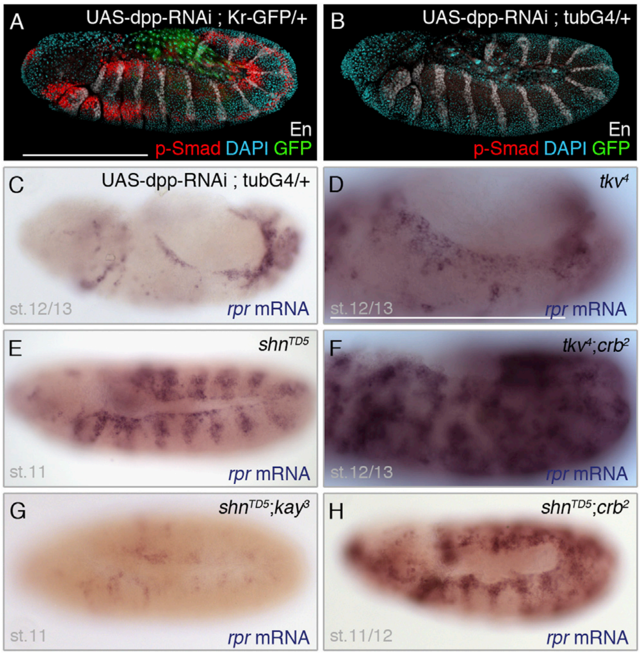

Figure 2. Dpp Signaling Counteracts JNK-Dependent Activation of *reaper* via the Transcriptional Repressor Schnurri

(A and B) p-Smad immunoreactivity, seen in control embryos (A), is not detectable in embryos carrying both UAS-dpp[RNAi] and *tubulin-Gal4* (B), confirming the effectiveness of this RNAi transgene. Anti-Engrailed and DAPI were used as morphological landmarks. En, Engrailed; st., stage.

(C) In embryos expressing UAS-dpp[RNAi] under the control of *tubulin-Gal4*, *reaper* expression becomes upregulated at the dorsal edge of the epidermis. Such embryos are often misshapen probably because of interference with the patterning activity of Dpp.

(D) Expression of *reaper* is also upregulated at the dorsal edge of homozygous *thickveins (tkv)* mutants.

(E) Upregulation of *reaper* in the dorsal and lateral epidermis of *schnurri* mutants. Expression can be seen along the whole dorsal edge but is segmental elsewhere. The basis of the segmental pattern is unknown.

(F) Upregulation of *reaper* throughout most of the epidermis of *thickveins crumbs* double mutants.

(G) No *reaper* upregulation is detected in *schnurri kayak (fos)* double mutants, demonstrating the requirement for canonical JNK signaling in *reaper* activation.

(H) Expression of *reaper* is upregulated throughout the epidermis of *schnurri crumbs* double mutants in a pattern that is roughly the sum of those seen in the single mutants.

Scale bar, 200 μm.

These observations suggested that loss of apicobasal polarity triggers JNK signaling (through an unknown mechanism), which, in turn, causes *reaper* expression and, hence, apoptosis. However, in the dorsal epidermis, JNK signaling does not activate *reaper* expression. Here, we show the molecular mechanism underpinning such protection and thus explain how JNK can control cell migration without triggering apoptosis.

RESULTS AND DISCUSSION

Activation of *reaper* by Canonical JNK Signaling Mediates Apoptosis in Response to Epithelial Disruption

In *crb* mutant embryos, *reaper* is strongly upregulated (Figures 1C–1F) in a pattern similar to that of apoptosis (Figures S1A and S1B available online), while the other two main proapoptotic genes, *hid* and *grim*, remain largely silent (Kolahgar et al., 2011)). No activated caspase immunoreactivity was detectable in *rpr*[87] *crb* double mutant embryos (Figure S1C), confirming the essential role of *reaper* and highlighting the need to uncover the mechanisms that activate *reaper* expression following loss of apicobasal polarity. As suggested previously (Kolahgar et al., 2011), JNK signaling is likely involved. Indeed, JNK signaling, as measured with a transcriptional reporter (AP-1 > GFP) (Chatterjee and Bohmann, 2012) was strongly activated in *crumbs* mutant embryos (Figures 1G–1J; Movie S1). Moreover, little *reaper* transcription was detectable in *crumbs* embryos that also lack *jra* or *kayak*, which encode the two components of AP-1, Jun, and Fos (Figures 1K–1N; see Figures S1F–S1I for *reaper* expression in the single mutants), and this was associated with a near-absence of apoptosis, as reported by activated caspase-3 immunoreactivity (Figures S1D and S1E). Notably, JNK signaling does not seem to necessarily cause apoptosis. In the ventral epidermis, the patterns of *reaper* expression and apoptosis (highlighted with anti-activated caspase) appeared to mirror the early segmental activation of JNK signaling (compare Figure 1D with Figure S1B), suggesting a relatively straightforward, likely causal, relationship there. However, neither *reaper* expression nor apoptosis were significantly activated in the dorsal epidermis, even at the dorsal edge, where JNK signaling is particularly active both in wild-type and *crumbs* mutants. What is the mechanism that protects the dorsal epidermis from the proapoptotic effect of JNK?

Dpp Signaling Prevents JNK from Activating *reaper* Expression in the Dorsal Epidermis

One feature of the dorsal epidermis is that it is under the influence of Dpp, a member of the BMP family of secreted growth factors (Hamaratoglu et al., 2014). Indeed, phospho-Smad (p-Smad) immunoreactivity (a mark of Dpp signaling) (Tanimoto et al., 2000) was detectable in this region before and during the time when JNK is active (Figure 2A). Therefore, Dpp signaling could prevent JNK signaling from activating *reaper* expression both in the dorsal epidermis of *crumbs* mutant embryos and, physiologically, at the dorsal edge of wild-type embryos. This was tested in embryos expressing a Dpp RNA interference (RNAi)-encoding transgene (Supplemental Information) under the control of the ubiquitous *tubulin-gal4* driver. This led to reduced signaling as indicated by the loss of p-Smad immunoreactivity from stage 11 onward (Figure 2B). In these embryos, a band of *reaper* transcription was observed at the dorsal edge (Figure 2C), where JNK is known to be activated in the wild-type (see Figure 1I). A similar result was seen in embryos lacking zygotic (but not maternal) activity of *thickveins* (*tkv*), which encodes an essential Dpp receptor (Nellen et al., 1994) (Figure 2D). Expression of *reaper* was also seen in the approximately ten-cell-wide dorsal region in *tkv crumbs* double mutants (as well as in the rest of the epidermis; Figure 2F), suggesting that Dpp signaling prevents *reaper* expression throughout the dorsal epidermis. Caspase immunoreactivity became detectable throughout the epidermis of *tkv crumbs* mutants (Figure S1J), consistent with the notion that repression of *reaper* expression by Dpp signaling translates into anti-apoptotic activity.

Schnurri Prevents *reaper* Transcription and Apoptosis

Dpp signaling is mediated by the Mad complex, which can activate or repress target genes depending on the sequence context and other cofactors (Affolter and Basler, 2007). The best characterized instance of repression by Dpp signaling is that of the *brinker* gene, which occurs via silencer elements where the Mad complex recruits the corepressor Schnurri (Affolter and Basler, 2007; Pyrowolakis et al., 2004). We therefore asked if Schnurri could mediate the repression of *reaper* by Dpp. Indeed, *reaper* transcription was upregulated at the dorsal edge of *schnurri* mutant embryos (Figure 2E; Figures S1O–S1R). In these embryos, *reaper* expression was also seen to extend segmentally in the lateral region, a feature not seen in Dpp-RNAi-expressing embryos (compare Figure 2E with Figure 2C), perhaps because Schnurri also has Dpp-independent activity, as suggested by (Kelsey et al., 2012). In any case, the *reaper*-repressive role of Schnurri in the dorsal epidermis was confirmed in *schnurri crumbs* double mutants, where *reaper* transcription was strongly upregulated in both the dorsal and ventral regions (Figure 2H). No *reaper* upregulation was seen in *schnurri kayak (fos)* double mutants (Figure 2G), showing that JNK signaling is an essential positive input to *reaper* transcription at the dorsal edge, as well as in segmentally repeated lateral domains, where the JNK sensor appears not to be sufficiently sensitive to detect activity. It is worth noting that staining of *schnurri* and *schnurri crumbs* embryos with anti-activated caspase confirmed that the repression of *reaper* transcription by Schnurri is needed to suppress apoptosis (Figures S1K and S1L). As AP-1 and Schnurri are both transcriptional regulators, we next sought to address whether the opposing influences of Schnurri and JNK signaling converge directly on the *reaper* promoter.

Binding Sites Upstream of the *reaper* Promoter Integrate the Effects of Dpp and JNK

The promoter region of *brinker* that mediates Schnurri-dependent repression has been extensively characterized, and mutation analysis identified an essential 16-base-pair (bp) repressor sequence (Pyrowolakis et al., 2004). Similar elements are found at ~350 positions in the *Drosophila* genome, defining a consensus sequence: GRCGNCNNNNNGTCTG (Pyrowolakis et al., 2004). Two related sites were identified upstream of *reaper*, in a region that is conserved in the 12 sequenced *Drosophila* species. In all these species, the proximal site (SE_p) is flanked on either side by a predicted AP-1-binding site, making it a potential regulatory element. Because SE_p is not an exact match to the consensus Schnurri binding site, we used an electrophoretic mobility shift assay (EMSA) to test whether it is recognized by Schnurri, using the previously characterized site from the *brinker* gene (cSE) (Pyrowolakis et al., 2004) as a positive control. Recombinant Schnurri protein induced a supershift of the Mad-Medea-DNA complex in both cases, although to a lesser extent with SE_p than with cSE (Figure S2A). Thus, we conclude that SE_p is recognized by Schnurri/Mad/Medea and could therefore mediate the repressive influence of Dpp on *reaper* expression. By extension, the module comprising SE_p and the two putative AP-1 sites could integrate the influence of Dpp and JNK signaling on *reaper* expression. To test this hypothesis in vivo, we made a reporter construct comprising 5.5 kb of sequence including this module and the basal *reaper* promoter, upstream of a GFP complementary DNA (Figure 3A). This reporter (*rpr-GFP*) and the variants described later were introduced by PhiC31-mediated integration at the same genomic location to allow comparison without confounding influence from position effects. The wild-type reporter was essentially silent in wild-type embryos (Figure 3B), as expected, since *reaper* expression is barely detectable during normal embryogenesis. By contrast, in *crumbs* mutants, *rpr-GFP* became segmentally upregulated in the ventrolateral—but not dorsal—epidermis (double-headed arrow in Figure 3C), thus mirroring the activity of the endogenous *reaper* gene in this background (compare to Figure 1F). Critically, like the endogenous *reaper* gene, this reporter became active in the dorsal epidermis of *schnurri* mutants (Figure 3D). Such dorsal expression was segmentally modulated (see Figure S2B for GFP staining alone), resembling the pattern of endogenous *reaper* transcription in *schnurri* mutants (Figure 2E). Also, like endogenous *reaper*, the reporter was widely and strongly activated in the epidermis of *schnurri crumbs* double mutants (Figure 3E). These observations suggest that Schnurri mediates repression of the reporter. To test the contribution of the predicted Schnurri binding site, a mutation was introduced in the reporter (ATCGTCTCGCC GTCTG → ATCGTCTCGCTTTCTG), thus creating *rpr[ΔShn]-GFP* (Figure 3A). This mutation was found to abrogate formation of the Mad-Medea-DNA complex in vitro (see SEm in Figure S2A), suggesting that *rpr[ΔShn]-GFP* would no longer be subject to repression by Schnurri. Indeed, this transgene became activated in wild-type embryos in the dorsal epidermis (Figure 3F). One must point out that this dorsal activity of *rpr[ΔShn]-GFP* did not appear before stage 13, 1–2 hr later than the appearance of *rpr-GFP* in *schnurri* mutants (compare Figure 3F to Figure 3D). Also, unlike *rpr-GFP* in *schnurri* mutants, *rpr[ΔShn]-GFP* was active in segmentally repeated ventral domains at stage 13. Despite these differences (see further discussion in the legend of Figure S2), the upregulation of *rpr-GFP* in *schnurri* mutants and the expression of *rpr[ΔShn]-GFP* in the dorsal epidermis of otherwise wild-type embryos (where *rpr-GFP* is silent) are consistent with the notion that Schnurri represses *reaper* expression in the dorsal epidermis, thus allowing JNK signaling to control epithelial migration without triggering apoptosis.

We next assessed the function of the two predicted AP-1 binding sites by mutating them (TGACTCATA → TGACATTTA) (Chatterjee and Bohmann, 2012) individually or as a pair in the GFP reporter (Figure 3A) and assessing their activity in the ventral epidermis of *crumbs* embryos, where the wild-type reporter is strongly activated. Mutating one or the other site reduced activation, while the double mutant reporter (*rpr[ΔAP1^P;ΔAP1^D]-GFP*) had no detectable activity in *crumbs* mutants (Figures 3H–3J; wild-type background in Figure S2E). This reporter is nevertheless functional. It was activated by irradiation (Figure 3K), consistent with the presence of a p53-response element previously shown to mediate the response to irradiation (Brodsky et al., 2000). It is worth pointing out here that *reaper* expression was still activated in *crb p53$^{5A\text{-}1\text{-}4}$* double mutants (P.F. Langton and J.-P.V., unpublished data), indicating that the response to loss of epithelial integrity does not require p53. Overall, our results show that the two predicted AP-1 binding sites contribute redundantly to *reaper* upregulation in *crumbs* mutants. They

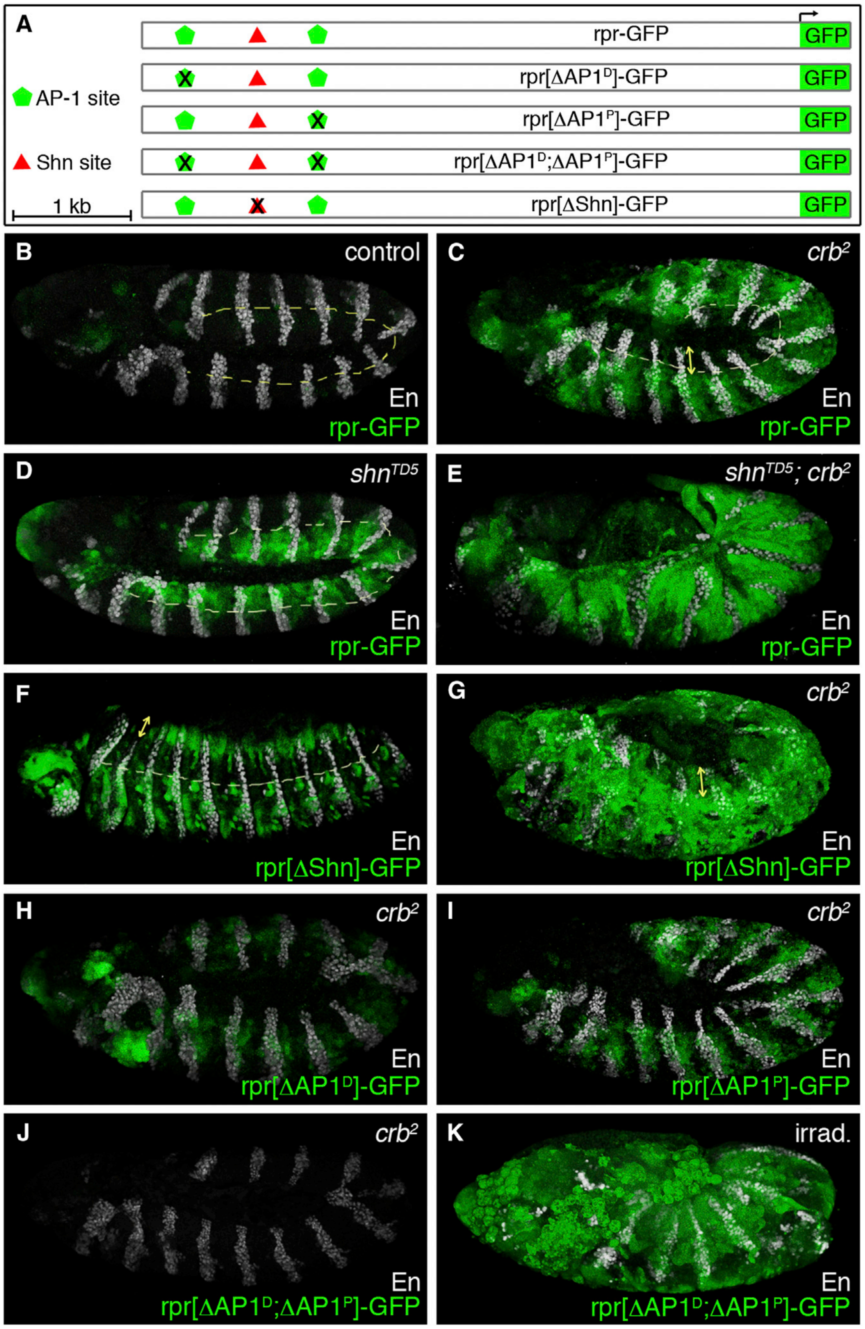

Figure 3. The *reaper* Promoter Integrates Inputs from JNK and Dpp Signaling

(A) Diagram of the reporter constructs that were tested in transgenic embryos; 5.5 kb of the *reaper* promoter were used to create *rpr-GFP*. Predicted binding sites for Schnurri or AP-1 were mutated to generate the variants listed. Details of the mutations are indicated in the text.

(B) The unmutated reporter (*rpr-GFP*) is almost silent in wild-type embryos. En, Engrailed.

(C) In *crumbs* mutant embryos, the same reporter is active in the ventrolateral, but not the dorsal (double-headed arrow), epidermis.

(D) By contrast, in *schnurri* mutants, *rpr-GFP* becomes active in the dorsal epidermis, suggesting that Schnurri is an essential repressive factor.

(E) Embryos lacking both *crumbs* and *schnurri* upregulate *rpr-GFP* throughout much of the epidermis.

(F) Mutation of the predicted Schnurri binding site leads to upregulation of the reporter in the dorsal epidermis wild-type embryos at stage 13.

(G) In *crumbs* mutant embryos, *rpr[ΔShn]-GFP* upregulation is seen in the dorsal epidermis (double-headed arrow) as well as in the ventrolateral epidermis, consistent with the notion that Schnurri contributes to preventing *reaper* expression in the dorsal epidermis of *crumbs* embryos.

(H–J) Upregulation of the *reaper* reporter in *crumbs* mutants requires the two predicted AP-1 binding sites. Deletion of either site leads to reduced expression while the double mutant reporter (*rpr[ΔAP1^D; ΔAP1^P]-GFP*) is silent.

(K) Gamma-irradiated embryos carrying the *rpr [ΔAP1^D; ΔAP1^P]-GFP* transgene express GFP throughout, showing that this reporter is functional.

All embryos are shown at stage 11 except for (F), which shows a stage 12–13 embryo. Engrailed (En), shown in white, provides an indication of the embryos' overall morphology.

also show that JNK signaling acts directly on the *reaper* promoter and not via a relay mechanism. We conclude that a small regulatory module allows JNK to trigger apoptosis, except in dorsal cells that are protected by Dpp signaling.

Schnurri Ensures the Survival of Dorsal Edge Cells during Dorsal Closure

JNK and Dpp signaling have extensively been shown to orchestrate dorsal closure. So far, attention has been focused on the role of these pathways in triggering the cell shape changes required for the dorsal epidermis to spread over the amnioserosa and meet at the dorsal midline (Fernández et al., 2007; Homsy et al., 2006; Riesgo-Escovar et al., 1996). Our results suggest an additional role for Dpp signaling during dorsal closure, namely, to ensure the survival of leading edge cells. We propose that such a protective mechanism is needed because of the proapoptotic influence of JNK signaling. Accordingly, the "dorsal open phenotype" of *schnurri* mutants (and possibly other Dpp pathway mutants) would not only be caused by the failure of dorsal edge cells to migrate but also by their reduced survival. To evaluate the contribution of the latter, we assessed the extent of tissue loss in fixed and live *schnurri* mutant embryos, which, as previously reported (Arora et al., 1995; Grieder et al., 1995; Staehling-Hampton et al., 1995), do not undergo dorsal closure. Cursory observation suggests that, in *schnurri* mutant embryos, the dorsal hole that appears at the end of germband retraction gapes open over time (Figures 4A and 4B; Figures S1Q and S1R). This was confirmed by confocal live imaging of *schnurri* mutants carrying *spider-GFP* as a marker of cell outlines

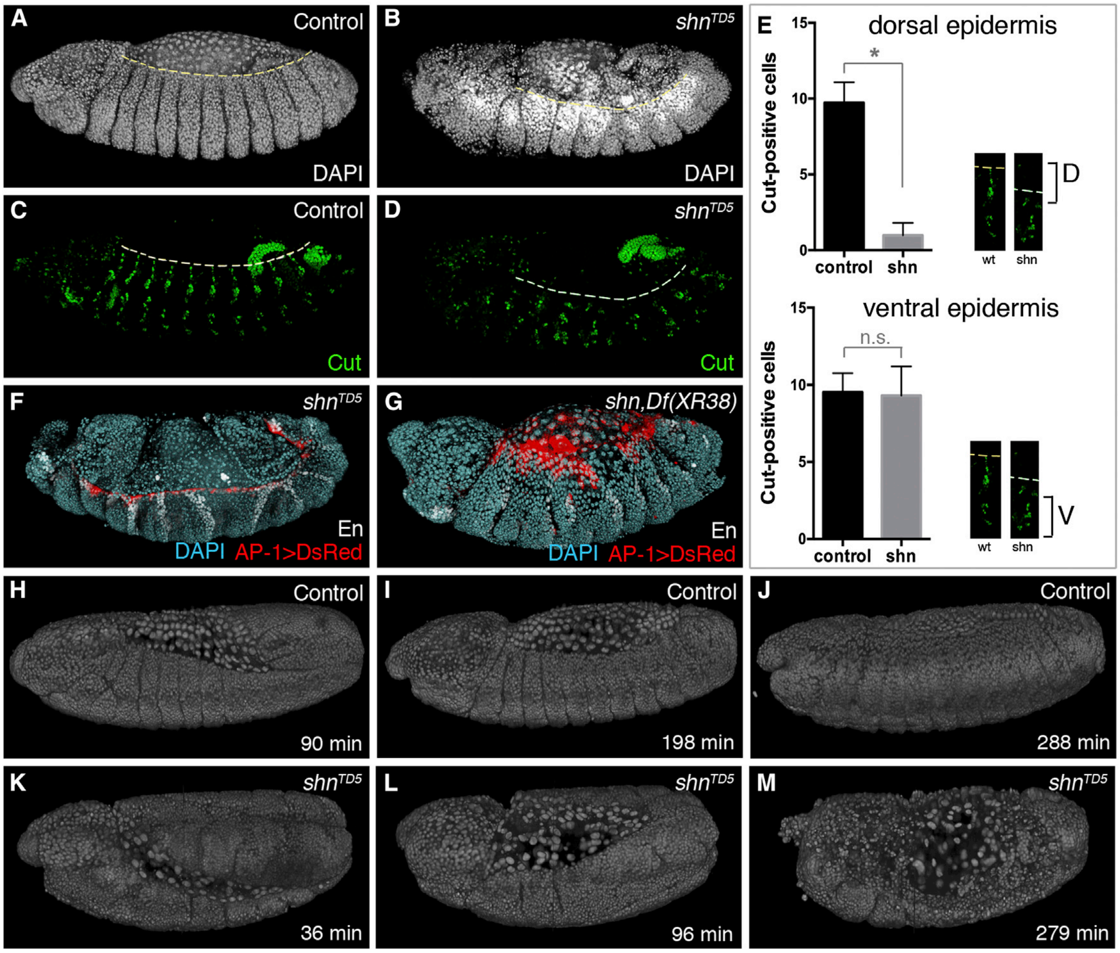

Figure 4. Dorsal Closure and Epithelial Loss in Wild-Type and *schnurri* Mutant Embryos

(A and B) Homozygous *schnurri* mutant embryos fail to achieve dorsal closure. A heterozygous embryo of similar stage is shown as control.

(C and D) The reduced number of Cut-positive cells in the dorsal cluster of *schnurri* mutant embryos suggests that the dorsal epidermis shrinks during the time when dorsal closure takes place in control embryos.

(E) Number of Cut-positive cells in the dorsal (D) and ventral (V) clusters of *schnurri* homozygotes and control heterozygotes at stage ~13. A significant loss of cells is seen in the dorsal cluster (Student's t test, *p < 0.0001). WT, wild-type; n.s., not significant. Error bars indicate SEM.

(F and G) *schnurri* and *schnurri reaper* mutant embryos carrying the JNK reporter. Gaping of dorsal hole is more pronounced in *schnurri* mutant (F) than in *schnurri reaper* mutant (G). En, Engrailed.

(H–M) Still images from MuVi-SPIM recordings (before, during, and after closure). Approximate times from the beginning of germband retraction are shown to allow comparison between the mutant and control samples. Control (H–J) and *schnurri* mutant (K–M) embryos carrying histone-RFP as a nuclear marker are shown. While dorsal cells migrate over the amnioserosa in the wild-type, these cells are progressively lost in *schnurri* mutants.

See also Movies S3 and S4.

(Figure S3D; Movie S2). To test if gaping of the dorsal hole could be due to tissue shrinkage, stage ~13 *schnurri* mutant embryos were stained with anti-Cut, which marks dorsal and ventral clusters of peripheral neurons along the D-V axis (Figures 4A–4D; Figures S3A–S3C), and the number of Cut-positive cells in each cluster was counted. While cell number in the dorsal cluster remained constant in wild-type embryos, it decreased significantly in *schnurri* mutants (Figure 4E). The number of ventral cells was relatively unaffected in both genotypes. It appears, therefore, that the dorsal epidermis is preferentially eliminated prior to this stage. Next, we used multiview light-sheet microscopy (MuVi-SPIM; Krzic et al., 2012) to achieve in toto imaging of *schnurri* mutants and control embryos carrying histone-red fluorescent protein (histone-RFP) (Figures 4H–4M). In *schnurri* mutants, cell debris could be seen around the dorsal edge, and many macrophages scurried around (Movies S3 and S4). Moreover, the number of epidermal nuclei became reduced, compared to that in control embryos (Figure S4). These observations confirm that, during stages 12–14, the dorsal epidermis of *schnurri* mutants progressively shrinks (Figures S4A–S4I), likely by apoptosis (see also Figures S1K, S1Q, and S1R). Consistent with the involvement of apoptosis, the dorsal hole did not appear to gape open in *schnurri* mutants that also lacked *reaper* (Figure 4G), even though these embryos failed to complete dorsal closure. As shown in Figure 4H, the JNK reporter remains active throughout tissue shrinkage (Figures 4F–4G), suggesting that, as

dorsal edge cells are eliminated, adjacent epidermal cells activate JNK. Taken together, our results suggest that the dorsal open phenotype of *schnurri* mutants is a combined consequence of tissue loss by apoptosis and lack of cell migration over the amnioserosa.

Most functions of Dpp signaling are achieved through Schnurri-dependent repression of the transcriptional repressor Brinker. However, as we have shown, inhibition of *reaper* by Schnurri is direct and, hence, likely Brinker independent. By contrast, the migration of dorsal edge cells is mediated by Brinker repression, since *schnurri brinker* double mutant larvae have a sealed dorsal midline (Marty et al., 2000; Torres-Vazquez et al., 2001). It is interesting that the dorsal epidermis of these double mutants is much reduced in surface area compared to that of wild-type embryos or *brinker* single mutant embryos, which have expanded dorsal fates (in Torres-Vazquez et al., 2001, compare Figure 2D to Figures 2A and 2B). We suggest that *schnurri brinker* double mutant embryos complete dorsal closure despite tissue loss, perhaps because a sufficient number of cells are able to migrate before undergoing apoptosis. To assess directly whether Brinker has any impact on *reaper* expression, we performed gain- and loss-of-function experiments (Figures S3E–S3I). No ectopic *reaper* expression was seen in embryos lacking or overexpressing *brinker* (Figures S3G and S3H). Moreover, Brinker overexpression did not prevent *reaper* expression in *crumbs* mutant embryos (Figures S3E and S3F), while overexpressing Schnurri did (Figures S3E and S3I). We conclude that Brinker does not affect *reaper* expression and that Schnurri contributes to dorsal closure through two parallel routes: first, by repressing Brinker and, hence, allowing the derepression of genes regulating the cytoskeletal functions required for cell migration (Fernández et al., 2007; Homsy et al., 2006); and second, by repressing *reaper*, thus ensuring survival of the dorsal edge cells in the face of JNK's proapoptotic pressure. Our results highlight the central role of Dpp and Schnurri in cell survival, extending observations on Dpp in imaginal discs (Adachi-Yamada et al., 1999; Gibson and Perrimon, 2005; Moreno et al., 2002; Shen and Dahmann, 2005) and TGFβ in vertebrates (Sabapathy et al., 1999; Taya et al., 1999).

Conclusions

In many contexts, JNK signaling leads to apoptosis, perhaps a remnant of this pathway's ancestral function in stress response (Ríos-Barrera and Riesgo-Escovar, 2013). JNK signaling may have started to regulate cytoskeletal functions in multicellular organisms to facilitate delamination or extrusion of defective cells. It is conceivable that such a regulatory relation might have subsequently been coopted to control other migratory behaviors such as those required for dorsal closure. Evidently, this would have necessitated coevolution of a protective, anti-apoptotic mechanism. At the dorsal edge of the epidermis, this is mediated by Dpp, which, intriguingly, is itself under the control of JNK signaling. Therefore, under the right circumstances, JNK contributes to the mechanism that counteracts its own AP-1-dependent proapoptotic pressure. The outcome of the regulatory network linking JNK and Dpp to *reaper* must be finely balanced, since a mild increase in JNK signaling (e.g., in *puckered* heterozygous embryos) triggers *reaper* expression (Kolahgar et al., 2011). Unlike the ventral epidermis, the dorsal epidermis seems prone to expressing Dpp in response to JNK, perhaps because of earlier expression in this region or through the action of additional regulators. This is likely to ensure the survival of dorsal edge cells during their migration. As we have shown, the opposing influences of Dpp and JNK are played out within the regulatory region of *reaper.* The anti-apoptotic activity of Dpp is mediated by Schnurri, a protein that could have more general anti-apoptotic activity since mammalian Schnurri has been shown to dampen cell death during T cell development (Staton et al., 2011). The function of this or similar regulatory modules in other tissues, contexts, and models can thus yield a broader understanding of the balance between apoptosis and survival at the intersection of signaling pathways.

EXPERIMENTAL PROCEDURES

Details on materials and methods can be found in the Supplemental Information. These include a full list of the *Drosophila* strains and antibodies as well as step-by-step staining protocols for immunofluorescence and in situ hybridization. For gamma irradiation, embryos were collected for 4 hr, aged a further 4 hr, and introduced in a gamma-cell irradiator for a 4,000 rad exposure (Nordstrom et al., 1996). The embryos were allowed to recover for 2 hr at 25°C before further analysis. The *reaper* reporter constructs were created by standard molecular biology with primers listed in the Supplemental Information. They were introduced into the *Drosophila* genome by PhiC31-mediated integration into PBac[yellow[+]-attP-9A] VK00027 (Bloomington *Drosophila* Stock Center # 9744, on chromosome III). EMSAs were largely performed as described elsewhere (Pyrowolakis et al., 2004). Live embryo imaging was performed either by classical confocal or MuVi-SPIM. For classical confocal microscopy, we used a Leica SP5 microscope equipped with a resonant scanner and a 20× (NA, 0.8) water immersion objective. For MuVi-SPIM, we used a custom-built set-up (two Nikon 10× 0.3-NA illumination objective lenses and two Nikon 25× 1.1-NA detection lenses) and protocols as described elsewhere (Krzic et al., 2012). The custom-modified Hamamatsu Flash 4 cameras were operated in the Lightsheet Readout Mode to reject scattered photons. Details on image processing and data visualization are provided in the Supplemental Information.

SUPPLEMENTAL INFORMATION

Supplemental Information includes Supplemental Experimental Procedures, four figures, and four movies and can be found with this article online at http://dx.doi.org/10.1016/j.devcel.2014.08.015.

ACKNOWLEDGMENTS

We thank the Bloomington Drosophila Stock Center for fly stocks, as well as Dirk Bohmann, Henri Jasper, Markus Affolter, and Kristin White for reagents and Alex Gould and Ingrid Poernbacher for discussions. We also thank Pedro Gaspar for help generating the reporters. Marvin Albert provided an early version of the Mercator projection routine. This work was funded by the UK's Medical Research Council (U117584268 to J.-P.V.) and a Wellcome Trust PhD Fellowship to J.V.B. S.G. was funded by the EMBL Interdisciplinary Postdoctoral Programme under Marie Curie Actions. L.H. acknowledges support from the Center of Modeling and Simulation in the Biosciences of the University of Heidelberg. Work in G.P.'s lab was supported by the research training program GRK1104 and the Excellence Initiative of the German Federal and State Governments (EXC294).

Received: October 30, 2013
Revised: June 27, 2014
Accepted: August 11, 2014
Published: October 9, 2014

REFERENCES

Adachi-Yamada, T., Fujimura-Kamada, K., Nishida, Y., and Matsumoto, K. (1999). Distortion of proximodistal information causes JNK-dependent apoptosis in Drosophila wing. Nature *400*, 166–169.

Affolter, M., and Basler, K. (2007). The Decapentaplegic morphogen gradient: from pattern formation to growth regulation. Nat. Rev. Genet. *8*, 663–674.

Arora, K., Dai, H., Kazuko, S.G., Jamal, J., O'Connor, M.B., Letsou, A., and Warrior, R. (1995). The Drosophila schnurri gene acts in the Dpp/TGF beta signaling pathway and encodes a transcription factor homologous to the human MBP family. Cell *81*, 781–790.

Bergmann, A., Agapite, J., McCall, K., and Steller, H. (1998). The Drosophila gene hid is a direct molecular target of Ras-dependent survival signaling. Cell *95*, 331–341.

Bogoyevitch, M.A., and Kobe, B. (2006). Uses for JNK: the many and varied substrates of the c-Jun N-terminal kinases. Microbiol. Mol. Biol. Rev. *70*, 1061–1095.

Brodsky, M.H., Nordstrom, W., Tsang, G., Kwan, E., Rubin, G.M., and Abrams, J.M. (2000). Drosophila p53 binds a damage response element at the reaper locus. Cell *101*, 103–113.

Chatterjee, N., and Bohmann, D. (2012). A versatile ΦC31 based reporter system for measuring AP-1 and Nrf2 signaling in Drosophila and in tissue culture. PLoS ONE *7*, e34063.

Chen, F. (2012). JNK-induced apoptosis, compensatory growth, and cancer stem cells. Cancer Res. *72*, 379–386.

De Smaele, E., Zazzeroni, F., Papa, S., Nguyen, D.U., Jin, R., Jones, J., Cong, R., and Franzoso, G. (2001). Induction of gadd45beta by NF-kappaB downregulates pro-apoptotic JNK signalling. Nature *414*, 308–313.

Dhanasekaran, D.N., and Reddy, E.P. (2008). JNK signaling in apoptosis. Oncogene *27*, 6245–6251.

Fernández, B.G., Arias, A.M., and Jacinto, A. (2007). Dpp signalling orchestrates dorsal closure by regulating cell shape changes both in the amnioserosa and in the epidermis. Mech. Dev. *124*, 884–897.

Gibson, M.C., and Perrimon, N. (2005). Extrusion and death of DPP/BMP-compromised epithelial cells in the developing Drosophila wing. Science *307*, 1785–1789.

Glise, B., and Noselli, S. (1997). Coupling of Jun amino-terminal kinase and Decapentaplegic signaling pathways in Drosophila morphogenesis. Genes Dev. *11*, 1738–1747.

Grieder, N.C., Nellen, D., Burke, R., Basler, K., and Affolter, M. (1995). Schnurri is required for Drosophila Dpp signaling and encodes a zinc finger protein similar to the mammalian transcription factor PRDII-BF1. Cell *81*, 791–800.

Hamaratoglu, F., Affolter, M., and Pyrowolakis, G. (2014). Dpp/BMP signaling in flies: From molecules to biology. Semin. Cell Dev. Biol. *32C*, 128–136.

Homsy, J.G., Jasper, H., Peralta, X.G., Wu, H., Kiehart, D.P., and Bohmann, D. (2006). JNK signaling coordinates integrin and actin functions during Drosophila embryogenesis. Dev. Dyn. *235*, 427–434.

Hou, X.S., Goldstein, E.S., and Perrimon, N. (1997). Drosophila Jun relays the Jun amino-terminal kinase signal transduction pathway to the Decapentaplegic signal transduction pathway in regulating epithelial cell sheet movement. Genes Dev. *11*, 1728–1737.

Igaki, T. (2009). Correcting developmental errors by apoptosis: lessons from Drosophila JNK signaling. Apoptosis *14*, 1021–1028.

Kelsey, E.M., Luo, X., Brückner, K., and Jasper, H. (2012). Schnurri regulates hemocyte function to promote tissue recovery after DNA damage. J. Cell Sci. *125*, 1393–1400.

Kolahgar, G., Bardet, P.-L., Langton, P.F., Alexandre, C., and Vincent, J.-P. (2011). Apical deficiency triggers JNK-dependent apoptosis in the embryonic epidermis of Drosophila. Development *138*, 3021–3031.

Krzic, U., Gunther, S., Saunders, T.E., Streichan, S.J., and Hufnagel, L. (2012). Multiview light-sheet microscope for rapid in toto imaging. Nat. Methods *9*, 730–733.

Leppä, S., and Bohmann, D. (1999). Diverse functions of JNK signaling and c-Jun in stress response and apoptosis. Oncogene *18*, 6158–6162.

Luo, X., Puig, O., Hyun, J., Bohmann, D., and Jasper, H. (2007). Foxo and Fos regulate the decision between cell death and survival in response to UV irradiation. EMBO J. *26*, 380–390.

Marty, T., Müller, B., Basler, K., and Affolter, M. (2000). Schnurri mediates Dpp-dependent repression of brinker transcription. Nat. Cell Biol. *2*, 745–749.

McEwen, D.G., and Peifer, M. (2005). Puckered, a Drosophila MAPK phosphatase, ensures cell viability by antagonizing JNK-induced apoptosis. Development *132*, 3935–3946.

Moreno, E., Basler, K., and Morata, G. (2002). Cells compete for decapentaplegic survival factor to prevent apoptosis in Drosophila wing development. Nature *416*, 755–759.

Nellen, D., Affolter, M., and Basler, K. (1994). Receptor serine/threonine kinases implicated in the control of Drosophila body pattern by decapentaplegic. Cell *78*, 225–237.

Nordstrom, W., Chen, P., Steller, H., and Abrams, J.M. (1996). Activation of the reaper gene during ectopic cell killing in Drosophila. Dev. Biol. *180*, 213–226.

Papa, S., Zazzeroni, F., Bubici, C., Jayawardena, S., Alvarez, K., Matsuda, S., Nguyen, D.U., Pham, C.G., Nelsbach, A.H., Melis, T., et al. (2004). Gadd45 beta mediates the NF-kappa B suppression of JNK signalling by targeting MKK7/JNKK2. Nat. Cell Biol. *6*, 146–153.

Pyrowolakis, G., Hartmann, B., Müller, B., Basler, K., and Affolter, M. (2004). A simple molecular complex mediates widespread BMP-induced repression during Drosophila development. Dev. Cell *7*, 229–240.

Riesgo-Escovar, J.R., and Hafen, E. (1997). Drosophila Jun kinase regulates expression of decapentaplegic via the ETS-domain protein Aop and the AP-1 transcription factor DJun during dorsal closure. Genes Dev. *11*, 1717–1727.

Riesgo-Escovar, J.R., Jenni, M., Fritz, A., and Hafen, E. (1996). The Drosophila Jun-N-terminal kinase is required for cell morphogenesis but not for DJun-dependent cell fate specification in the eye. Genes Dev. *10*, 2759–2768.

Ríos-Barrera, L.D., and Riesgo-Escovar, J.R. (2013). Regulating cell morphogenesis: the Drosophila Jun N-terminal kinase pathway. Genesis *51*, 147–162.

Sabapathy, K., Jochum, W., Hochedlinger, K., Chang, L., Karin, M., and Wagner, E.F. (1999). Defective neural tube morphogenesis and altered apoptosis in the absence of both JNK1 and JNK2. Mech. Dev. *89*, 115–124.

Shaulian, E., and Karin, M. (2002). AP-1 as a regulator of cell life and death. Nat. Cell Biol. *4*, E131–E136.

Shen, J., and Dahmann, C. (2005). Extrusion of cells with inappropriate Dpp signaling from Drosophila wing disc epithelia. Science *307*, 1789–1790.

Staehling-Hampton, K., Laughon, A.S., and Hoffmann, F.M. (1995). A Drosophila protein related to the human zinc finger transcription factor PRDII/MBPI/HIV-EP1 is required for dpp signaling. Development *121*, 3393–3403.

Staton, T.L., Lazarevic, V., Jones, D.C., Lanser, A.J., Takagi, T., Ishii, S., and Glimcher, L.H. (2011). Dampening of death pathways by schnurri-2 is essential for T-cell development. Nature *472*, 105–109.

Stronach, B.E., and Perrimon, N. (1999). Stress signaling in Drosophila. Oncogene *18*, 6172–6182.

Tanimoto, H., Itoh, S., ten Dijke, P., and Tabata, T. (2000). Hedgehog creates a gradient of DPP activity in Drosophila wing imaginal discs. Mol. Cell *5*, 59–71.

Taya, Y., O'Kane, S., and Ferguson, M.W. (1999). Pathogenesis of cleft palate in TGF-beta3 knockout mice. Development *126*, 3869–3879.

Torres-Vazquez, J., Park, S., Warrior, R., and Arora, K. (2001). The transcription factor Schnurri plays a dual role in mediating Dpp signaling during embryogenesis. Development *128*, 1657–1670.

Weston, C.R., and Davis, R.J. (2007). The JNK signal transduction pathway. Curr. Opin. Cell Biol. *19*, 142–149.

White, K., Grether, M.E., Abrams, J.M., Young, L., Farrell, K., and Steller, H. (1994). Genetic control of programmed cell death in Drosophila. Science *264*, 677–683.

Developmental Cell, Volume *31*

Supplemental Information

The Dpp/TGFβ-Dependent Corepressor Schnurri Protects Epithelial Cells from JNK-Induced Apoptosis in *Drosophila* Embryos

Jorge V. Beira, Alexander Springhorn, Stefan Gunther, Lars Hufnagel, Giorgos Pyrowolakis, and Jean-Paul Vincent

Figure S1

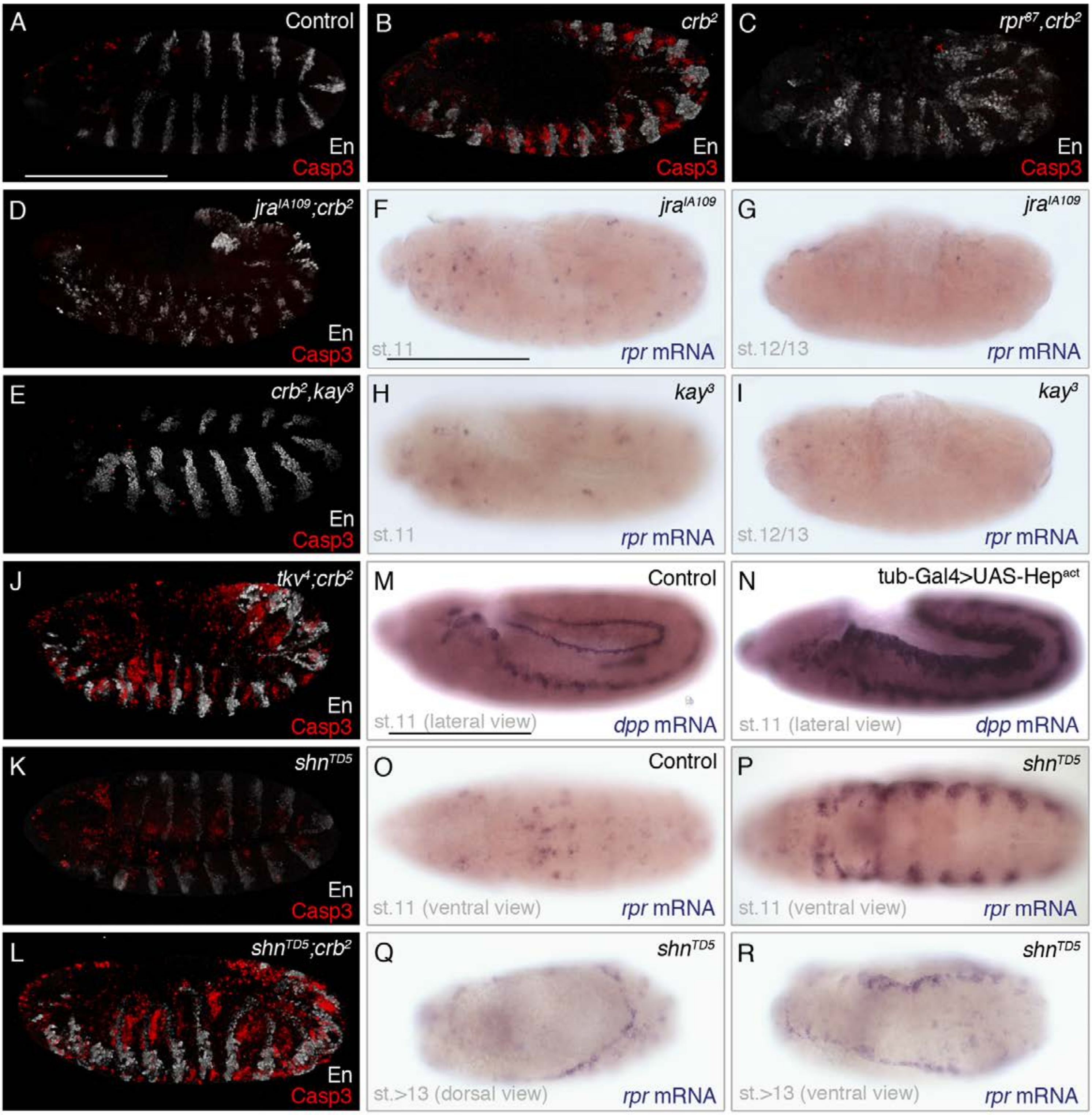

A
Control
En
Casp3
B
crb2
En
Casp3
C
rpr87,crb2
En
Casp3
D
jraIA109;crb2
En
Casp3
F
jraIA109
st.11
rpr mRNA
G
jraIA109
st.12/13
rpr mRNA
E
crb2,kay3
En
Casp3
H
kay3
st.11
rpr mRNA
I
kay3
st.12/13
rpr mRNA
J
tkv4;crb2
En
Casp3
M
Control
st.11 (lateral view)
dpp mRNA
N
tub-Gal4>UAS-Hepact
st.11 (lateral view)
dpp mRNA
K
shnTD5
En
Casp3
O
Control
st.11 (ventral view)
rpr mRNA
P
shnTD5
st.11 (ventral view)
rpr mRNA
L
shnTD5;crb2
En
Casp3
Q
shnTD5
st.>13 (dorsal view)
rpr mRNA
R
shnTD5
st.>13 (ventral view)
rpr mRNA

SUPPLEMENTAL FIGURES AND LEGENDS

Figure S1 (related to Figure 1). The apoptotic response to epithelial disruption is mediated by *reaper* and modulated by Schnurri. (A-B) Occasional apoptotic cells, marked with anti-Caspase-3, can be seen in control embryos (A) while apoptosis is substantially increased in the ventral epidermis of *crumbs* mutants (B). (**C**) Anti-Caspase-3 staining shows that only a small number of apoptotic cells appear in rpr^{87}*crumbs* double mutants. (**D-E**) Staining of double mutants also show that *jun (jra)* and *fos (kayak)* are required for apoptosis in *crumbs* mutants. (**F-I**) Absence of *reaper* expression in *jun* (*jra*) or *fos* (*kay*) single mutants, as seen in *jun crumbs* and *crumbs kayak* double mutants shown in Fig. 1. (**J-L**) Widespread apoptosis in the epidermis of *tkv crumbs* double mutants (**J**). Ectopic apoptosis in the dorsal epidermis of *shn* mutants (**K**). Caspase3 immunoreactiviy is seen throughout the epidermis of *shn crumbs* double mutants (**L**), including in the dorsal epidermis, which is protected in *crumbs* single mutants. (**M**) Expression of *dpp* in a control embryo (*tubulin-gal4/*+). (**N**) Over-expression of Hemipterous, Drosophila JNKK (*tubulin-gal4/+, UAS-Hep/*+), leads to excess transcription of *dpp*. (**O-P**) Expression of *reaper* is upregulated in the dorso-lateral epidermis of stage 11 *schnurri* mutant embryos (P). Compare to control embryo (O, *shn[TD5]/+)*. Ventral views are used to show the lateral extent of expression. (**Q-R**) At later stages (>13) a band of expression remains at the dorsal edge of the epidermis. Dorsal view at this stage shows the gaping hole (R), while ventral view shows all the epidermis that remains.

Figure S2

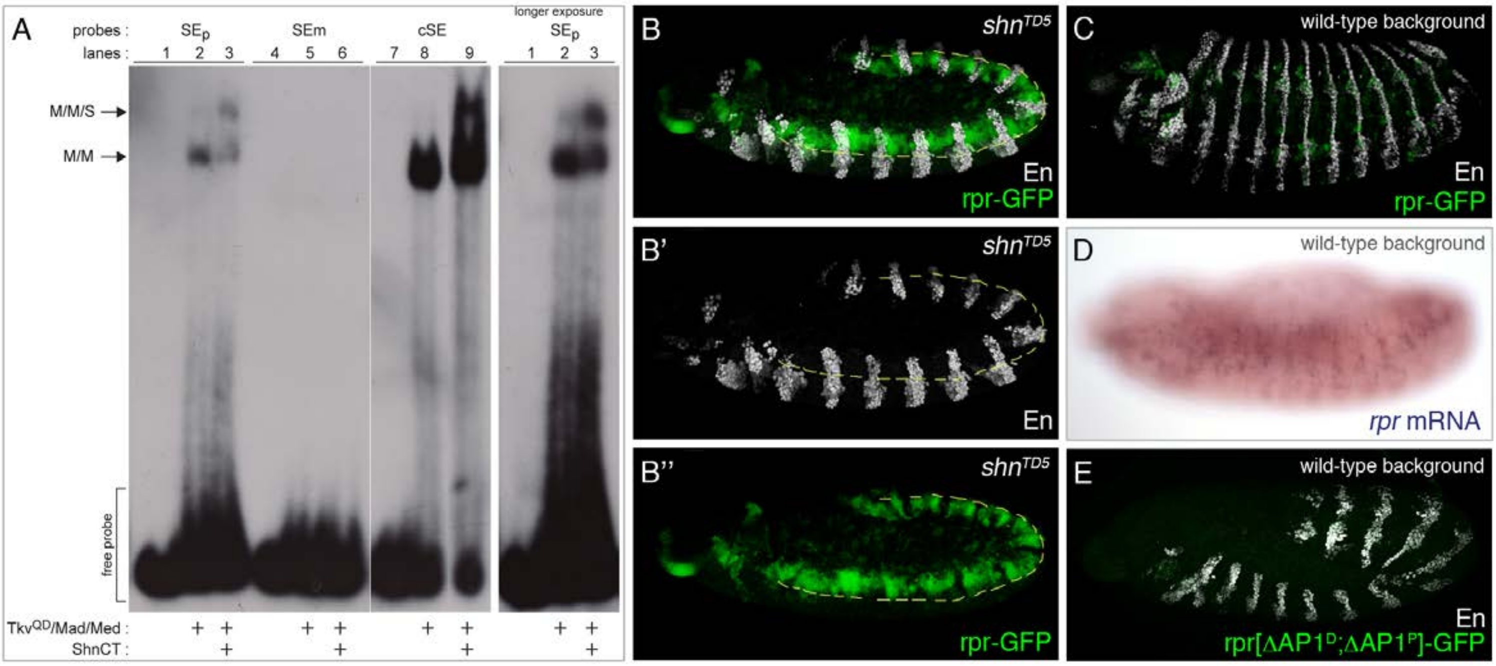

A
probes :
SEp
SEm
cSE
longer exposure
SEp
lanes :
1 2 3
4 5 6
7 8
9
1 2 3
M/M/S
M/M
free probe
TkvQD/Mad/Med :
ShnCT :
B
shnTD5
En
rpr-GFP
B'
shnTD5
En
B''
shnTD5
rpr-GFP
C
wild-type background
En
rpr-GFP
D
wild-type background
rpr mRNA
E
wild-type background
En
rpr[ΔAP1D;ΔAP1P]-GFP

Figure S2 (related to Figure 2). JNK and Dpp signaling regulate *reaper* expression directly on the promoter. (**A**) Binding of the Schnurri/Mad/Medea complex as detected by EMSA, on the proximal silencer element (SE_p) from the *reaper* promoter (referred to as SE_p in the text). No binding can be detected on the mutated element (SEm; same mutation as in the *rpr[ΔShn]-GFP* transgenic reporter). The previously characterized SE from *brinker* is used as a positive control (cSE). The indicated probes were incubated with extracts from S2 cells transfected with constitutively active Thickveins (TkvQD), Mad and Medea (to generate pMad/Med complexes (M/M)) and with an N-terminally truncated version of Schnurri (ShnCT) (to generate pMad/Medea/ Schnurri complexes (M/M/S)). (**B**) Expression of the *rpr-GFP* reporter in *schnurri* mutants (like in Fig. 3D) is segmentally modulated; separate channels for GFP (B'') and Engrailed (B') are shown. (**C-D**) At later stages (stage 13, 14 and later), *rpr-GFP* has a segmental expression in ventral groups of cells of wild-type embryos (C), similarly to endogenous *reaper* at these later stages (D). (**E**) Weak expression of *rpr[ΔAP1^P;ΔAP1^D]-GFP* in an otherwise wild-type background (compare to Fig. 2J and 2B). As mentioned in the main text, expression of *rpr[ΔShn]-GFP* in wild type embryos is delayed relative to that of *rpr-GFP* in *schnurri* mutants. Timely activation of *reaper* expression in the dorsal epidermis could conceivably require an enhancer that is missing in the reporter. Alternatively, it is possible that loss of Schnurri not only removes *reaper* repressor activity but may also, in addition, indirectly allow increased JNK activity in the dorsal epidermis, thus accounting for early activation of *rpr-GFP*. It is also worth noting the segmental expression of *rpr[ΔShn]-GFP* at stage 13 (in addition to the expected dorsal expression). This may not be directly attributable to loss of Schnurri since, at this late stage, similar though weaker expression was also detectable from the *rpr-GFP* transgene as well as from the endogenous *reaper* gene in wild type embryos (Fig. S2C,D).

Figure S3

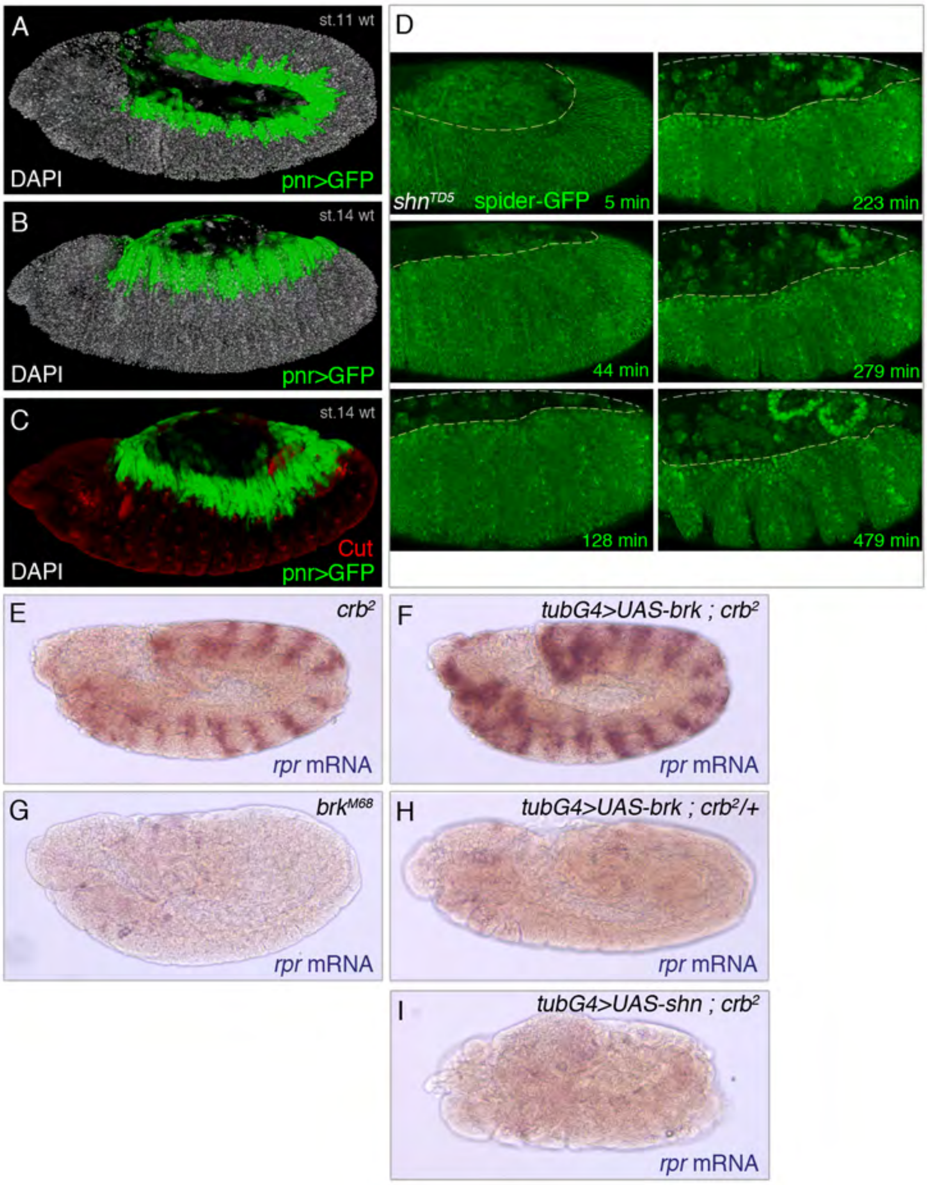

A
st.11 wt
DAPI
pnr>GFP
B
st.14 wt
DAPI
pnr>GFP
C
st.14 wt
DAPI
Cut
pnr>GFP
D
shnTD5 spider-GFP
5 min
223 min
44 min
279 min
128 min
479 min
E
crb2
rpr mRNA
F
tubG4>UAS-brk ; crb2
rpr mRNA
G
brkM68
rpr mRNA
H
tubG4>UAS-brk ; crb2/+
rpr mRNA
I
tubG4>UAS-shn ; crb2
rpr mRNA

Figure S3 (related to Figure 3). The dorsal epidermis is reduced in the absence of Schnurri. (**A-B**) Expression of *pannier* (as indicated in *pannier-gal4 UAS-GFP*), a gene activated by Dpp signaling (Winick et al., 1993) marks the dorsal epidermis, where Dpp signaling is active. (**C**) Position of the dorsal and ventral clusters of Cut expression (highlighted with anti-Cut) relative to the pannier domain (see Fig. 4). (**D**) Stills from a confocal movie of a *schnurri* mutant embryo expressing spider-GFP as a marker of cell outlines. Over time, epidermal cells appear to be lost from the dorsal side, progressively exposing the gut. Many macrophages are seen to scurry around the dorsal edge (see movie S2). (**E-H**) Expression of *reaper* following alterations in *brinker* and *schnurri* activity. *brinker* loss of function does not result in ectopic *reaper* (G). Brinker overexpression did not prevent *reaper* upregulation in *crumbs* mutants (F), while overexpression of Schnurri did (I). Moreover, Brinker overexpression had no detectable effect on *reaper* expression in control embryos (H).

Figure S4

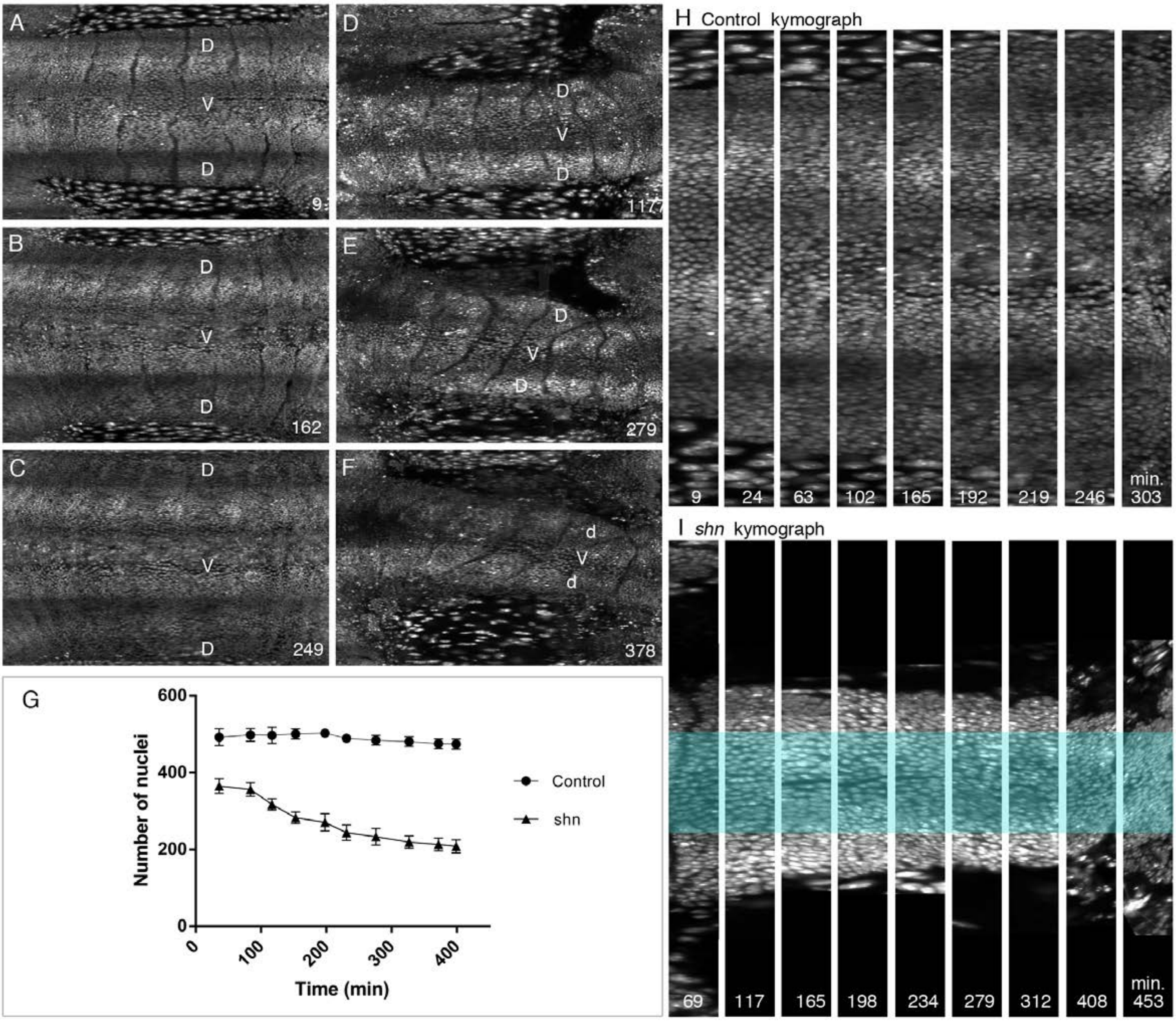

A
D
V
D
9
B
D
V
D
162
C
D
V
D
249
D
D
V
D
117
E
D
V
D
279
F
d
V
d
378
G
600
400
200
0
Number of nuclei
0
100
200
300
400
Time (min)
Control
shn
H Control kymograph
9
24
63
102
165
192
219
246
min.
303
I shn kymograph
69
117
165
198
234
279
312
408
min.
453

Figure S4 (related to Figure 4). Dorsal closure defects in the absence of Schnurri. (A-I) *In toto* live imaging of control and *schnurri* mutants using MuVi-SPIM. The epidermis was computationally projected on a single plane (see Supplemental Materials and Methods below) and is displayed with the ventral midline (V) horizontally in the middle. (**A-C**) In wild type embryos, migration of epidermal cells progressively covers the amnioserosa recognizable by its large nuclei (top and bottom of each frame). By contrast, dorsal closure fails in *schnurri* mutants (amnioserosa remains uncovered; **D-F**), and the dorsal epidermis (D) becomes reduced (see also movies S3 and S4). (**G**) The number of histone-RFP nuclei per segment (counted in the three central segments (7-9)) decreased over time in *schnurri* mutants, while remaining mostly constant in control embryos. (**H-I**) Such reduction is also apparent on a kymograph displaying segment 8 at selected time points. Cell debris are also seen around the dorsal edge (see also movies S3 and S4), as expected from increased apoptosis there.

SUPPLEMENTAL MOVIE LEGENDS

Movie S1 (related to Figure 1). Activity of TRE-GFP, a reporter of JNK activity, in an otherwise wild type embryo (left) or a *crumbs* mutant embryo (right) from stage 10/11 onwards. In the wild type, GFP fluorescence can be seen at the dorsal edge during dorsal closure while in the mutant widespread fluorescence becomes detectable as development proceeds. In this and in movie S2, embryos are oriented with anterior on the left and dorsal at the top.

Movie S2 (related to Figure 4). Dorsal closure in embryos carrying spider-GFP to mark cell outlines (lateral view showing dorsal closure at the top). Dorsal closure proceeds normally in the control embryo (left, *schnurri* heterozygote) but is impaired in the *schnurri* mutant (on the right). Note progressive shrinkage of the dorsal epidermis in the *schnurri* mutant during this period.

Movie S3 (related to Figure 4). Whole-embryo imaging (MuVi-SPIM) of a wild-type (control) followed by a *shn* mutant embryo (second half of the movie) carrying histone-RFP. Initially, eight different views, each rotated by 45 degrees, are shown to highlight distinct view points around the embryo, starting with a dorsal view (followed by lateral, ventral, lateral on subsequent views). Subsequent frames show development during and after dorsal closure. Anterior is to the centre of the rosette made up by the eight views. The first half of the movie shows the wild-type (control) embryo, while the second half shows a *shn* mutant, first from the dorsal side and then every 45 degrees. Observation over time and from several angles confirms the progressive loss of epithelial tissue from the dorsal edge, where some cell debris can be seen and where macrophages later congregate.

Movie S4 (related to Figure 4). Control (top) and *schnurri* mutant (bottom) embryos at three representative time points (before, during and after dorsal closure). Each embryo undergoes a

360 degrees rotation around the A-P axis to allow viewing of the whole epidermis (generated from MuVi-SPIM data). This shows the progressive loss of epidermal cells in the mutant, while dorsal cells migrate normally over the amnioserosa in the wild-type.

SUPPLEMENTAL MATERIALS AND METHODS

Drosophila genetics. Most alleles used on this study were balanced over fluorescently marked balancer chromosomes in order to distinguish homozygous mutant embryos from their heterozygous counterparts, which were used as controls. Balancer chromosomes used include: CTG (CyO, Twist-Gal4>UAS-GFP), CKG (CyO, Krüppel-Gal4>UAS-GFP), TTG (TM3, Sb, Ser, Twist-Gal4>UAS-GFP), TDY (TM6, Hu, Sb, Deformed-YFP) and TKG (TM3, Sb, Krüppel-Gal4>UAS-GFP). The following fly stocks were used: *crb*^{2}*/TM6* (BDSC #3448). *crb*^{2}*/TTG. w;;tub-Gal4,crb*^{2}*/TM3. UAS-CD8-GFP, crb*^{2}*/TDY. Def(3L)XR38/TDY. shn*^{TD5}*/Cyo-wgLacZ. shn*^{TD5}*/CTG. TRE-GFP;crb*^{2}*/TDY,* JNK signaling reporter from (Chatterjee and Bohmann, 2012). *jra*^{IA109}*/CTG* (BDSC #3273). *kay*^{3}*/TTG* (from Henri Jasper, (Biteau and Jasper, 2011)). *jra*^{IA109}*/CKG;crb*^{2}*/TTG. kay*^{3}*,crb*^{2}*/TTG. rpr*^{87}*/TM2* (Tan et al., 2011). *rpr*^{87}*,crb*^{2}*/TTG. shn*^{TD5}*/CKG;crb*^{2}*/TTG. shn*^{TD5}*/CKG;kay*^{3}*/TTG. tub-Gal4/TKG. UAS-Hep*^{act} (from Dirk Bohmann). *UAS-dppRNAi* (BDSC #36779) (Ni et al., 2011). *tkv*^{4}*/CTG. tkv*^{4}*/CKG;crb*^{2}*/TTG. shn*^{TD5}*/CKG;spider-GFP. shn*^{TD5}*/CyO; histone-GFP* (or his-RFP). *rpr-GFP. shn*^{TD5}*/CKG;rpr-GFP. shn*^{TD5}*,TRE-DsRed/CKG;rpr-GFP. rpr-GFP,crb*^{2}*/TTG. shn*^{TD5}*/CKG;rpr-GFP,crb*^{2}*/TTG. rpr[ΔShn]-GFP. rpr[ΔShn]-GFP,crb*^{2}*/TTG. rpr[ΔAP1*^{P}*]-GFP. rpr[ΔAP1*^{P}*]-GFP*;*crb*^{2}*/TTG. rpr[AP1*^{D}*]-GFP. rpr[ΔAP1*^{D}*]-GFP*;*crb*^{2}*/TTG. rpr[ΔAP1*^{P}*;ΔAP1*^{D}*]-GFP. rpr[ΔAP1*^{P}*;ΔAP1*^{D}*]-GFP*;*crb*^{2}*/TTG.*

Immunofluorescence and in situ hybridization. Embryos were fixed in 6% formaldehyde/heptane interface for 20 min and then devitellinised with methanol. They were

stored in methanol until further processing. In situ hybridisation to a digoxigenin-labelled single stranded RNA probe was performed at 63°C without proteinase K (Kolahgar et al., 2011). When necessary, hybridized embryos were stained with anti-GFP to confirm the genotype. Hybridized embryos were mounted in 1:1 glycerol / PBS photographed with a Zeiss Axiophot-2 microscope coupled to a Zeiss AxioCam HRC camera and the Axiovision Rel. 4.7 software. Immunofluorescence was performed according to standard protocols. The following primary antibodies were used: mouse anti-Engrailed (mAb4D9, Developmental Studies Hybridoma Bank (DSHB), 1/200), mouse anti-Wingless (mAb4D4, DSHB, 1/1000), mouse anti-p-Smad (mAb pS423/425, Epitomics, 1/200), chicken anti-GFP (Abcam, 1/1000), and chicken anti-β-Galactosidase (Abcam cat 13970-100; 1/1000). Appropriate secondary antibodies were obtained from Molecular Probes. Stained embryos were mounted in Vectashield (Vector laboratories) or glycerol 50% diluted in PBS and stored at 4 °C. Images were acquired using a Leica SP5 laser-scanning confocal microscope and assembled in ImageJ/Fiji (NIH), Photoshop (Adobe) or Volocity (Perkin Elmer).

Reporter constructs. The upstream region of *reaper* including the transcription start site (5.5 kb) was amplified from genomic DNA by PCR with the following primers: 5’-CACCCGGAAAATGGAAATGTAAG-3’ (forward) and 5’-ATGATTTTTTTCGAGATGCG-3’ (reverse) and cloned in front of a GFP cDNA. This ‘wild type’ reporter (*rpr-GFP*) was then used as a template for directed mutagenesis to generate the variant reporters described in the text. All the reporters were introduced into the *Drosophila* genome by PhiC31-mediated integration into PBac[yellow[+]-attP-9A] VK00027 (BDSC# 9744, on Chr. III). The transgenes were also inserted into PBac[yellow[+]-attP-3B] VK00002 (BDSC# 9723, on Chr. II), which gave similar (not shown) results.

EMSA. For electrophoretic mobility shift assays (EMSA), *Drosophila* S2 cells were either transfected with expression plasmids for Tkv^{QD} (50 ng), Mad (175 ng) and Med (175 ng) or with expression plasmids for ShnCT (400 ng). Cells were harvested 72 hr after transfection and lysed for 10 min at 4°C in 100 µl of 100 mM Tris (pH 7.5), 1 mM DTT, 0.5% TritonX100 and 1% NP40. Radioactively labeled probes were generated by annealing and filling in partially overlapping oligonucleotides in the presence of α-32 dATP. Binding reactions were carried out in a total volume of 25 µl containing 12.5 µl 2x binding buffer (200 mM KCl, 40 mM HEPES (pH 7.9), 40% glycerol, 2 mM DTT, 0.6% BSA and 0.02% NP40), 10000 cpm of radioactively labeled probe, 1 µl poly dIdC (1mM) and 7 µl of cleared S2 cell extracts. After incubation for 30 min at 4°C, the reactions were analyzed by non-denaturing 4% polyacrylamide gel electrophoresis followed by autoradiography (Pyrowolakis et al., 2004).

Live imaging by classical confocal microscopy. After dechorionation in 50% bleach for 2 minutes, embryos were washed with water. Live embryos of the appropriate stage and genotype were selected under a fluorescence microscope. They were then mounted on a thin strip of glue at the bottom of a Petri dish, covered with PBS, and imaged at 22°C in the Live Data mode of an upright Leica SP5 microscope equipped with a resonant scanner. A 20x (NA=0.8) water immersion objective (Leica) was used. Typical acquisition time was 40-55 seconds/embryo. The time-lapse series were assembled and analysed with ImageJ/Fiji.

Live imaging by MuVi-SPIM. Embryos were imaged with a custom-built MuVi-SPIM setup with two illumination objective lenses (Nikon 10x 0.3NA) and two detection lenses (Nikon 25x 1.1NA), as previously described (Krzic et al., 2012). Two custom-modified Hamamatsu Flash 4 cameras were used to record the opposing views of the sample. These cameras were operated in the Lightsheet Readout Mode™ to reject scattered photons (De Medeiros et al., 2014). Embryo

preparation and mounting in transparent gels was performed as previously described (Krzic et al., 2012). Every three minutes, two perpendicular oriented z-stacks (each with 200 planes and 1 μm spacing) covering the entire embryo were recorded. These stacks were computationally fused into a single 3D dataset for each time point. Slight shifts of the embryo in the gel over the 14hrs image period were compensated by a generic particle image velocimetry method written in Matlab (www.mathworks.com). Epidermis projections: for *in toto* analysis and visualization of development of the embryonic epidermis, the dimensionality of the entire dataset (3D images and time) was reduced by projecting the surface of the embryo to a plane; first, for each time point, we determined the convex hull of the 3D surface of the embryo based on the fluorescent intensity distribution in the 3D image. A standard Mercator projection was then applied to map the embryonic surface to a plane. Despite this projection having an effect on size and shape of objects at the embryo poles, the analysis was done in the central locations where it yields an unaltered representation of the tissue away from the poles. A multi-slice Mercator projection was generated, covering a thin layer around the embryos surface using Python (www.python.org). A secondary maximum projection of the multiple Mercator slices into a single 2D image provides a complete view of the fluorescent intensity distribution of the embryos surface, thus the epidermis. For the movies, volumetric rendering of the 3D fluorescent intensity distributions were performed using Fiji (www.fiji.sc) with the 3D-Viewer plugin. The rendering yields a 2D view of the fluorescence distribution as observed from a viewpoint (e.g. dorsal and ventral) outside of the embryo. Viewpoints and time points were rendered independently and then stitched together.

SUPPLEMENTAL REFERENCES

Biteau, B., and Jasper, H. (2011). EGF signaling regulates the proliferation of intestinal stem cells in Drosophila. Development *138*, 1045-1055.

Chatterjee, N., and Bohmann, D. (2012). A versatile ΦC31 based reporter system for measuring AP-1 and Nrf2 signaling in Drosophila and in tissue culture. PLoS ONE *7*, e34063.

De Medeiros, G.Q.G., Norlin, N., Gunther, S., Albert, M., Krzic, U., and Hufnagel, L. (2014). Multiview light-sheet microscopy goes confocal. *Under review*.

Kolahgar, G., Bardet, P.-L., Langton, P.F., Alexandre, C., and Vincent, J.-P. (2011). Apical deficiency triggers JNK-dependent apoptosis in the embryonic epidermis of Drosophila. Development *138*, 3021-3031.

Krzic, U., Gunther, S., Saunders, T.E., Streichan, S.J., and Hufnagel, L. (2012). Multiview light-sheet microscope for rapid in toto imaging. Nature methods *9*, 730-733.

Ni, J.-Q., Zhou, R., Czech, B., Liu, L.-P., Holderbaum, L., Yang-Zhou, D., Shim, H.-S., Tao, R., Handler, D., Karpowicz, P.*, et al.* (2011). A genome-scale shRNA resource for transgenic RNAi in Drosophila. Nature methods *8*, 405-407.

Pyrowolakis, G., Hartmann, B., Müller, B., Basler, K., and Affolter, M. (2004). A simple molecular complex mediates widespread BMP-induced repression during Drosophila development. Developmental cell *7*, 229-240.

Tan, Y., Yamada-Mabuchi, M., Arya, R., St Pierre, S., Tang, W., Tosa, M., Brachmann, C., and White, K. (2011). Coordinated expression of cell death genes regulates neuroblast apoptosis. Development *138*, 2197-2206.

Winick, J., Abel, T., Leonard, M.W., Michelson, A.M., Chardon-Loriaux, I., Holmgren, R.A., Maniatis, T., and Engel, J.D. (1993). A GATA family transcription factor is expressed along the embryonic dorsoventral axis in Drosophila melanogaster. Development *119*, 1055-1065.
